# *Trichomonas vaginalis* adhesion protein 65 facilitates human papillomavirus entry via SPCS1-mediated upregulation of CD151 and HSPG2 in keratinocyte lineage

**DOI:** 10.1186/s40249-025-01381-x

**Published:** 2025-11-06

**Authors:** Xuefang Mei, Wanxin Sheng, Yani Zhang, Wenjie Tian, Xiaowei Tian, Zhenke Yang, Shuai Wang, Zhenchao Zhang

**Affiliations:** Present Address: Xinxiang Key Laboratory of Pathogenic Biology, Department of Pathogenic Biology, School of Basic Medical Sciences, Xinxiang Medical University, Xinxiang, 453003 Henan People’s Republic of China

**Keywords:** *Trichomonas vaginalis*, Human papillomavirus, Adhesion protein 65, HPV membrane receptor molecules, Co-infection

## Abstract

**Background:**

Cervical cancer driven by human papillomavirus (HPV) infection remains a critical global health challenge. Co-infection with *Trichomonas vaginalis*, a prevalent sexually transmitted protozoan, is strongly associated with increased susceptibility to HPV, yet the molecular basis for this synergy is unclear. Here, we investigated the role of *T. vaginalis* adhesion protein 65 (TvAP65) in HPV entry, focusing on its interaction with host factors in epithelium.

**Methods:**

Using in vitro (human adult low calcium high temperature keratinocytes, HaCaT cells) and in vivo (BALB/c athymic nude mice, BALB/cA-nu mice) models, we assessed HPV infection rates and the expression of HPV entry receptors (cluster of differentiation 151, CD151 and heparan sulfate proteoglycan 2, HSPG2) under *T. vaginalis* exposure. TvAP65 was either knocked down or overexpressed to evaluate its functional impact. A siRNA screen targeting 12 host molecules that interact with TvAP65 identified signal peptidase complex subunit 1 (SPCS1) as a key mediator. Dual knockdown of TvAP65 and SPCS1 or HPV receptors (CD151/HSPG2) was performed to dissect mechanistic hierarchies. Statistical analyses were performed using Student’s *t*-test for two-group comparisons and analysis of variance (ANOVA) for comparisons involving three or more groups (*P* < 0.05).

**Results:**

*T. vaginalis* markedly enhanced HPV entry in epithelial cells by upregulating CD151 and HSPG2 (*P* < 0.001). TvAP65 knockdown reversed this effect, reducing HPV infection by 21.76 ± 0.12% (*P* < 0.001) and protein-level expression of the receptors (*P* < 0.001), while overexpression amplified both. Strikingly, SPCS1 knockdown alone attenuated HPV infection by 33.61 ± 0.40% and abolished *T. vaginalis*-driven CD151/HSPG2 upregulation. Dual knockdown of TvAP65 and SPCS1 synergistically suppressed HPV entry (54.64 ± 0.39% reduction, *P* < 0.001), confirming the central role of SPCS1 in TvAP65-mediated receptor activation.

**Conclusions:**

Our study unveils a previously uncharacterized mechanism by which *T. vaginalis* exacerbates HPV infection: TvAP65 hijacks SPCS1 to transcriptionally upregulate CD151 and HSPG2, thereby facilitating HPV entry into host cells. This *TvAP65-SPCS1-CD151/HSPG2* axis highlights potential therapeutic targets to disrupt the synergy between HPV and *T. vaginalis*, offering new strategies for cervical cancer prevention.

**Graphical Abstract:**

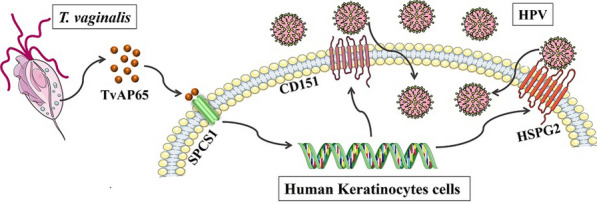

**Supplementary Information:**

The online version contains supplementary material available at 10.1186/s40249-025-01381-x.

## Background

*Trichomonas vaginalis* is one of the most common non-viral sexually transmitted pathogens, causing persistent reproductive tract infections [1]. According to the World Health Organization, there are an estimated 376 million new cases of curable sexually transmitted infections each year worldwide, including *T. vaginalis* [2]. However, due to the high proportion of asymptomatic cases and the low sensitivity of preferred diagnostic methods, a substantial number of infections remain undiagnosed [3, 4]. Furthermore, as trichomoniasis is not a notifiable disease, the true epidemiological burden is likely underestimated [5]. Recognizing its public health significance, the U.S. Centers for Disease Control and Prevention has listed trichomoniasis as one of the neglected parasitic infections [6].

Beyond localized inflammation, *T. vaginalis* infection is associated with a range of complications, including vaginitis, urethritis, cervicitis, prostatitis, and increased susceptibility to human immunodeficiency virus (HIV) infection and cancer development [7–9]. In pregnant women, infection increases the risk of preterm birth and low birth weight [10]. Despite being characterized as a relatively mild and treatable disease, trichomoniasis has a high global incidence, and growing resistance to metronidazole poses additional treatment challenges [11, 12].

Critically, epidemiological studies have shown that *T. vaginalis* as a significant co-factor in cervical carcinogenesis [13–16]. Cervical cancer causes over 660,000 new cases and 350,000 deaths in 2022 [17], predominantly driven by persistent infections with high-risk human papillomavirus (hrHPV) types, especially HPV16 and HPV18 [18]. *T. vaginalis* amplifies HPV16 infection risk by 6.5-fold and worsens cancer prognosis [13, 19]. Although inflammation-mediated epithelial disruption partially explains the elevated risk of HPV infection among *T. vaginalis*-positive women [19–21], this alone may not fully account for the dramatic increase in HPV susceptibility observed. Therefore, the impact of *T. vaginalis* on HPV infection remains an important public health issue.

As an extracellular pathogen, *T. vaginalis* relies on adhesion proteins, notably *T. vaginalis* adhesion protein 65 (TvAP65), to colonize host cells [22]. TvAP65, a multifunctional malic enzyme homolog, plays a pivotal role in parasite adhesion, modulation of host cell proliferation, and induction of apoptosis [23, 24]. Passive immunization or blockade of TvAP65 markedly attenuates *T. vaginalis* pathogenicity [24], underscoring its importance in host-parasite interactions. Crucially, whether TvAP65 modulates HPV entry receptors remains unexplored.

HPV entry into genital tract cells depends on the mediation of multiple cell membrane receptor molecules, including heparan sulfate proteoglycans (HSPGs), integrin alpha 6 (ITGA6), integrin beta 4 (ITGB4), cluster of differentiation 63 (CD63), and cluster of differentiation 151 (CD151) [25–28]. We hypothesized that *T. vaginalis* hijacks TvAP65 to upregulate these receptors via host interactors, thereby creating a permissive niche for HPV. Here, we identify signal peptidase complex subunit 1 (SPCS1), a signal peptide peptidase-like protease, as the critical host mediator linking TvAP65 to CD151/HSPG2 overexpression. Through in vitro assays, combinatorial siRNA knockdowns, and in vivo models, we delineate a *TvAP65-SPCS1-CD151/HSPG2* axis that drives HPV entry into cervical epithelium, unveiling actionable targets to disrupt this pathogenic synergy.

## Methods

### Ethics compliance

All animal experiments were reviewed and approved by the Ethics Review Committee of Xinxiang Medical College (Reference No. XYLL-20210184). Female BALB/c athymic nude mice (BALB/cA-nu mice), 6 weeks old and specific pathogen-free (SPF) grade, were housed under sterile conditions with ad libitum access to autoclaved feed and water. Humane endpoints were strictly observed; mice nearing death were euthanized by exposure to 60–70% CO_2_ for 5 min, and cervical dislocation was performed to confirm death when necessary.

### Culture conditions and experimental models

Cell culture: Human adult low calcium high temperature keratinocytes (HaCaT cells) were purchased from Procell Life Science & Technology Co., Ltd. (Wuhan, China) and maintained in dulbecco's modified eagle medium (DMEM; Procell Life Science & Technology Co., Ltd., Wuhan, China) supplemented with 10% fetal bovine serum (FBS, Procell Life Science & Technology Co., Ltd., Wuhan, China) and 1% penicillin-streptomycin at 37 °C in a humidified atmosphere containing 5% CO_2_. Cells were passaged upon reaching 70–80% confluency using 0.25% trypsin-EDTA for digestion.

*T. vaginalis* culture: The *T. vaginalis* used in this study was isolated from vaginal secretions of women presenting clinical symptoms of trichomoniasis. The strain was identified as actin genotype E by PCR-restriction fragment length polymorphism (PCR-RFLP) and has been maintained in our laboratory for long-term storage. Parasites strains were cultured in trypticase-yeast extract-maltose (TYM) medium (TUOPU Biol-engineering Co., Ltd., Shandong, China) supplemented with 20% FBS and 1% penicillin–streptomycin at 37 °C in 5% CO₂. Parasites in the logarithmic growth phase were passaged at a 1∶50 dilution into fresh medium.

In vitro* T. vaginalis* infection: HaCaT cells were plated in a 6-well plate, 1 × 10^6^ per well. After cells adhered, they were washed twice with pre-warmed phosphate-buffered saline (PBS, Procell Life Science & Technology Co., Ltd., Wuhan, China), and 1 × 10^6^ *T. vaginalis* and 2 ml DMEM medium (Procell Life Science & Technology Co., Ltd., Wuhan, China) were added per well and incubated at 37 °C in a humidified atmosphere containing 5% CO₂.

In vivo* T. vaginalis* infection: On the first and eighth days, 500 μg of estradiol valerate dissolved in 100 μl of sesame oil (Solarbio Science and Technology Co., Ltd., Beijing China) was subcutaneously injected into 6-week-old female BALB/c-nu mice. From days 6 to 9, intraperitoneally inject each mouse with dexamethasone (10 mg/kg, dissolved in 100 μl of PBS) daily for four consecutive days. On day 10, 1 × 10^6^ *T*. *vaginalis* trophozoite suspended in 10 μl of TYM medium (TUOPU Biol-engineering Co., Ltd., Shandong, China) were inoculated into the vaginal canal. To confirm persistent *T. vaginalis* infection, vaginal lavages were collected daily from days 1 to 8 post-inoculation and cultured in TYM medium for 24 h at 37 ℃. Parasite viability was assessed by measuring the optical density (OD), confirming sustained infection throughout the experimental period (Additional file 1: Fig. S1).

In vitro HPV infection: HaCaT cells were exposed to red fluorescent protein (RFP)-tagged HPV18 pseudovirus particles (Kemei Borui Technology Co., Ltd., Beijing, China) in DMEM at a final concentration of 5 × 10^5^ TU/ml.

In vivo HPV infection: On day 1, mice were pretreated with 100 μl of 30 mg/ml medroxyprogesterone acetate (Yuanye Bio-Technology Co., Ltd., Shanghai, China) via subcutaneous injection. On day 5, vaginal mucosa was gently abraded with a sterile cell brush, followed by intravaginal administration of 9 μl RFP-HPV18 pseudovirus (5 × 10^5^ TU/ml) mixed with 3 μl of 4% carboxymethylcellulose (CMC; Yuanye Bio-Technology Co., Ltd., Shanghai, China). The mixture was inoculated into the mouse vagina. After 24 h, mice were euthanized by exposure to 60–70% CO_2_ for 5 min, and vaginal tissues were collected for HPV infection analysis using an in vivo imaging system.

### RNA interference

RNA interference (RNAi) was employed to silence target genes. siRNAs targeting CD151 and HSPG2, along with corresponding negative controls (sc-42829, sc-44010, sc-37007), were purchased from Santa Cruz Biotechnology (Shanghai, China). siRNAs targeting TvAP65 and its interacting partners were designed and synthesized by Suzhou Jinweizhi Biotechnology (Suzhou, China). Three siRNA pairs were designed per gene, and the most efficient one was selected. These sequences were provided in Table 1. All RNAi experiments were performed based on three independent biological replicates, each conducted in triplicate wells.

**Table 1:** The siRNA sequences of TvAP65 and its interacting molecules

Name	Sequence（5’→3’）
TvAP65-siRNA	sense: CGUUGAUGCUGUCAAGGAATT antisense: UUCCUUGACAGCAUCAACGAA
TV-NC-siRNA	sense: CGUCCCGUAGCCCACUAAAdTdT antisense: UUUAGUGGGCUACGGGACGdTdT
FTH1-siRNA	sense: AGAUCAACCUGGAGCUCUATT antisense: UAGAGCUCCAGGUUGAUCUTT
SPCS1-siRNA	sense: CCUCUCAAGUGGUUACCUGUUTT antisense: AACAGGUAACCACUUGAGAGGTT
ATP5MC3-siRNA	sense: GCAGCUGUUCUCAUAUGCUAUTT antisense: AUAGCAUAUGAGAACAGCUGCTT
IGFBP7-siRNA	sense: GUCACUAUGGAGUUCAAAGGATT antisense: UCCUUUGAACUCCAUAGUGACTT
PMEPA1-siRNA	sense: GAGCAAAGAGAAGGAUAAACAT antisense: UGUUUAUCCUUCUCUUUGCUCTT
REEP5-siRNA	sense: CGGCGUGAACAGGAGCUUCAUTT antisense: AUGAAGCUCCUGUUCACGCCGTT
BNIP3-siRNA	sense: GAACUGCACUUCAGCAAUAAUTT antisense: AUUAUUGCUGAAGUGCAGUUCTT
C4orf3-siRNA	sense: CUAAUUUGCAGAACUCUAUUATT antisense: UAAUAGAGUUCUGCAAAUUAGTT
C6orf89-siRNA	sense: CCACUGGAAGGUCUACGUUAUTT antisense: AUAACGUAGACCUUCCAGUGGTT
GRAMD1A-siRNA	sense: GGAGCGGCAUUGAAGACUATT antisense: UAGUCUUCAAUGCCGCUCCTT
TNFRSF12A-siRNA	sense: GCUGACACUGACUAAGGAATT antisense: UUCCUUAGUCAGUGUCAGCTT
PKM-siRNA	sense: GAACUUUGCCAUGAAUGUUGG antisense: AACAUUCAUGGCAAAGUUCAC
NC-siRNA	sense: UUCUCCGAACGUGUCACGUdTdT antisense: ACGUGACACGUUCGGAGAAdTdT

*TV Trichomonas vaginalis*, *TvAP65 Trichomonas vaginalis* adhesion protein 65, *NC* negative control, *SPCS1* signal peptidase complex subunit 1

All other gene names (FTH1, ATP5MC3, IGBP7, PMEPA1, REEP5, BNIP3, C4orf3, C6orf89, GRAMD1A, TNFRSF12A, PKM) are official gene symbols according to the National Center for Biotechnology Information Gene database and are presented in their standard abbreviated form

For *T. vaginalis*, 5 × 10^5^ parasites were resuspended in 400 μl serum- and antibiotic-free TYM basal medium and seeded into 24-well plates. The RNAi procedure was adapted from previously reported protocols [22, 24] with slight modifications. Briefly, TvAP65-siRNA (5 μl) and 1.2 μl of Lipofectamine 2000 (Thermo Fisher Scientific, Waltham, MA, USA) were separately diluted in 50 μl Opti-MEM I (Thermo Fisher Scientific, Waltham, MA, USA), then combined after 5 min and incubated at room temperature for 20 min to form transfection complexes. These complexes were added to the parasite suspension and incubated at 37 °C with 5% CO₂. Afterward, 500 μl of complete TYM medium was added. Various siRNA concentrations (0–200 nmol/L) and incubation times (0–36 h) were tested to determine optimal transfection conditions, which were found to be 200 nmol/L siRNA for 24 h, as observed under fluorescence microscopy (Additional file 2: Fig. S2). Blank control, Lipofectamine-only, and negative control (NC-siRNA) groups were included. Gene silencing efficiency was verified by qPCR (primer sequences are listed in Table 2) and Western blotting.

**Table 2:** Sequences of primers used for qPCR

Name	Sequence（5’→3’）
TvAP65	Forward: CACGCAAGGATGCTGAGAGA Reverse: GAACACCAGAAACGCCGATG
Tv-Actin	Forward: TCACAGCTCTTGCTCCACCA Reverse: AAGCACTTGCGGTGAACGAT
HSPG2	Forward: TGAGTCCTTCTACTGGCAGC Reverse: ATGTTGTTGCCCGTGATCTG
CD151	Forward: ATGGGTGAGTTCAACGAGAAGA Reverse: GCAGGCTGATGTAGTCACTCT
ITGA6	Forward: ATGCACGCGGATCGAGTTT Reverse: TTCCTGCTTCGTATTAACATGCT
ITGB1	Forward: CAAGAGAGCTGAAGACTATCCCA Reverse: TGAAGTCCGAAGTAATCCTCCT
FTH1	Forward: GTCAGCTTAGCTCTCATCAC Reverse: ACGTCTATCTGTCTATGTCTTG
SPCS1	Forward: GTTTGCTGACACTTCCTCC Reverse: AATTTTTCTTTCCCCTGGT
ATP5MC3	Forward: TTCAGACCAGTGCAATCAG Reverse: AAAGCAACCATCAAACAAA
IGFBP7	Forward: CATCACCCAGGTCAGCAAG Reverse: TCACAGCTCAAGTACACCTG
PMEPA1	Forward: TGTCAGGCAACGGAATCCC Reverse: CAGGTACGGATAGGTGGGC
REEP5	Forward: GGGTAGTGTATGGTGTGT Reverse: TCTTCGCTTCTTTAGTGA
BNIP3	Forward: CAGCATGAGTCTGGACGGAG Reverse: GCCGACTTGACCAATCCCAT
C4orf3	Forward: AGAAAATAACCCTCTCCCC Reverse: TGAACCTACTCTTCCCAGC
C6orf89	Forward: TCATCCATCACATTAGGC Reverse: TCACAGTCGTTTGTCCAC
GRAMD1A	Forward: GATGCTCTCTTCTCGGACTCG Reverse: GATGGGGATGGTGTACGTC
TNFRSF12A	Forward: AGCACCTCCTGCCCCCTT Reverse: CCGCCGGTCTCCTCTATG
PKM	Forward: GACGGAGGTGGAAAATGGTG Reverse: CAGATGCCTTGCGGATGAAT
GAPDH	Forward: CGGAGTCAACGGATTTGGTCGTAT Reverse: AGCCTTCTCCATGGTGGTGAAGAC

*TV Trichomonas vaginalis*, *TvAP65 Trichomonas vaginalis* adhesion protein 65, *NC* negative control, *CD151* cluster of differentiation 151, *HSPG2* heparan sulfate proteoglycan 2, *ITGA6* integrin alpha 6, *ITGB1* integrin beta 1, *GAPDH* glyceraldehyde 3-phosphate dehydrogenase, *SPCS1* signal peptidase complex subunit 1

All other gene names (FTH1, ATP5MC3, IGBP7, PMEPA1, REEP5, BNIP3, C4orf3, C6orf89, GRAMD1A, TNFRSF12A, PKM) are official gene symbols according to the National Center for Biotechnology Information Gene database and are presented in their standard abbreviated form

For HaCaT cells, 1 × 10⁶ cells were seeded into 6-well plates containing 1.5 ml of Opti-MEM I. siRNAs (10 μl of 10 μmol/L for CD151/HSPG2; 10 μl of 20 μmol/L for other targets) were diluted in 250 μl of Opti-MEM I and mixed with 5 μl Lipofectamine 2000 pre-diluted in 250 μl of Opti-MEM I. After a 20 min incubation, the transfection complexes were added to the cells and incubated at 37 °C with 5% CO₂. Gene silencing efficiency was confirmed by qPCR (primer sequences are listed in Table 2) and Western blotting in both control and experimental groups (Additional file 3: Fig. S3; Additional file 4: Fig. S4).

### TvAP65 overexpression

To achieve overexpression of TvAP65, the pDsRed-N1-TvAP65 eukaryotic expression vector (2500 ng) was transfected into 1 × 10⁶ HaCaT cells using 10 μl of Lipofectamine 2000. Briefly, the plasmid and Lipofectamine were each diluted in 250 μl of Opti-MEM I reduced-serum medium, incubated separately for 5 min in the dark, then combined and incubated for 20 min to allow formation of transfection complexes. These complexes were added to HaCaT cells seeded in 6-well plates containing 1.5 ml of Opti-MEM I, and the cells were cultured at 37 °C with 5% CO₂. Control groups included blank controls and cells transfected with empty vector (pDsRed-N1) groups (*n* = 3 per group). TvAP65 expression was assessed by qPCR (primer sequences are listed in Table 2) and Western blotting (Additional file 5: Fig. S5).

### HPV in vitro infection assay

After 24 h of co-incubation with wild-type or TvAP65-knockdown *T. vaginalis*, or following genetic manipulation of HaCaT cells (TvAP65 overexpression, or silencing of TvAP65-interacting molecules, HSPG2, or CD151), cells were exposed to RFP-labeled HPV18 pseudovirus (5 × 10^5^ TU/ml) for 48 h. After washing with PBS, HPV infection efficiency was evaluated by fluorescence microscopy and flow cytometry.

To rule out apoptosis-related increases in infection, cell viability was determined using Trypan blue and Calcein acetoxymethyl ester (Calcein AM) staining. Calcein AM (Servicebio Technology Co., Ltd., Wuhan, China) was used according to the manufacturer’s instructions, and green fluorescent live cells were visualized under a fluorescence microscope.

### HPV in vivo infection assay

Six-week-old female BALB/cA-nu mice, 6 per group, were intravaginally infected with either wild-type or TvAP65-silenced *T. vaginalis*. After 24 h, RFP-labeled HPV18 pseudovirus was administered intravaginally. Another 24 h later, mice were euthanized using 60–70% CO_2_ for 5 min, and vaginal tissues were collected for HPV infection analysis using an in vivo imaging system. Control group mice received no treatment. HPV infection data were obtained from three independent experiments.

### Analysis of HPV membrane receptor expression

Total RNA and proteins were extracted from HaCaT cells infected with *T. vaginalis* or genetically modified (TvAP65-overexpressing or receptor-silenced cells). Expression levels of HPV receptors (HSPG2, CD151, ITGA6, ITGB1) were assessed by qPCR (primer sequences are listed in Table 2) and Western blot.

### Protein–protein interaction analysis

The amino acid sequence of SPCS1 (UniProt ID: Q9Y6A9) was retrieved from the UniProt database (https://www.uniprot.org/), and that of TvAP65 (TrichDB ID: TVAGG3_0979910) was obtained from the TrichDB database (https://trichdb.org/). Predicted 3D structures of both proteins were acquired from the AlphaFold Protein Structure Database and imported into PyMOL 3.0.4 (Schrödinger, New York, NY, USA) for structural analysis. The protein–protein interface was visualized, interacting residues and interatomic distances were identified and annotated, and docking results were presented as structural models after adjusting viewing angles.

For experimental validation, co-immunoprecipitation (Co-IP) was performed. Eukaryotic expression plasmids pDsRed-N1-Flag-SPCS1 and pCMV-3HA-TvAP65 were co-transfected into 293 T cells (60 mm dishes) using 12 μl Lipofectamine 2000. After 24 h, cells were harvested and washed with PBS. Co-IP was carried out using a commercial kit (Protein A + G magnetic beads, Beyotime Biotechnology, Shanghai, China) according to the manufacturer’s protocol. Briefly, 3.5 μg of affinity-purified anti-HA antibody (or mouse IgG as a control) was incubated with magnetic beads at 37 °C for 2 h. Subsequently, 350 μl of ice-cold IP lysis buffer and the protein lysate from one 100 mm culture dish were added, followed by gentle mixing at room temperature for 2 h. After washing, the bound proteins were eluted with 100 μl elution buffer and analyzed by Western blotting.

### Quantitative PCR

Total RNA was extracted with TRIzol reagent (Thermo Fisher Scientific, Waltham, MA, USA), and cDNA was synthesized with a reverse transcription kit (Takara Bio, Shiga, Japan). qPCR was performed with SYBR Green Master Mix (Takara Bio, Shiga, Japan) on a CFX96 Touch Real-Time PCR Detection System (Bio-Rad). Gene expression was normalized to Actin for *T. vaginalis* and to glyceraldehyde 3-phosphate dehydrogenase (GAPDH) for HaCaT cells, using the 2^−ΔΔCt^ method.

### Western blot analysis

Proteins were separated by Sodium Dodecyl Sulfate–Polyacrylamide Gel Electrophoresis and transferred to Polyvinylidene difluoride membranes. Membranes were blocked with 5% non-fat milk, incubated with primary antibodies overnight at 4 °C, and then with HRP-conjugated secondary antibodies. Signal detection was performed using enhanced chemiluminescence reagents (Thermo Fisher Scientific, Waltham, MA, USA) and band intensities were quantified using Image J software.

### Statistical analysis

Statistical analysis was performed using GraphPad Prism 9.0 (GraphPad Software, San Diego, CA, USA). All data were presented as mean ± standard deviation (SD) from at least three independent experiments. Statistical significance between two groups was determined using Student’s *t*-test, and comparisons among multiple groups were analyzed by one-way or two-way ANOVA followed by Tukey’s post hoc test for multiple comparisons. A two-tailed *P* < 0.05 was considered statistically significant.

## Results

### *T. vaginalis* enhances HPV infection in live epithelial cells

**Fig. 1:**
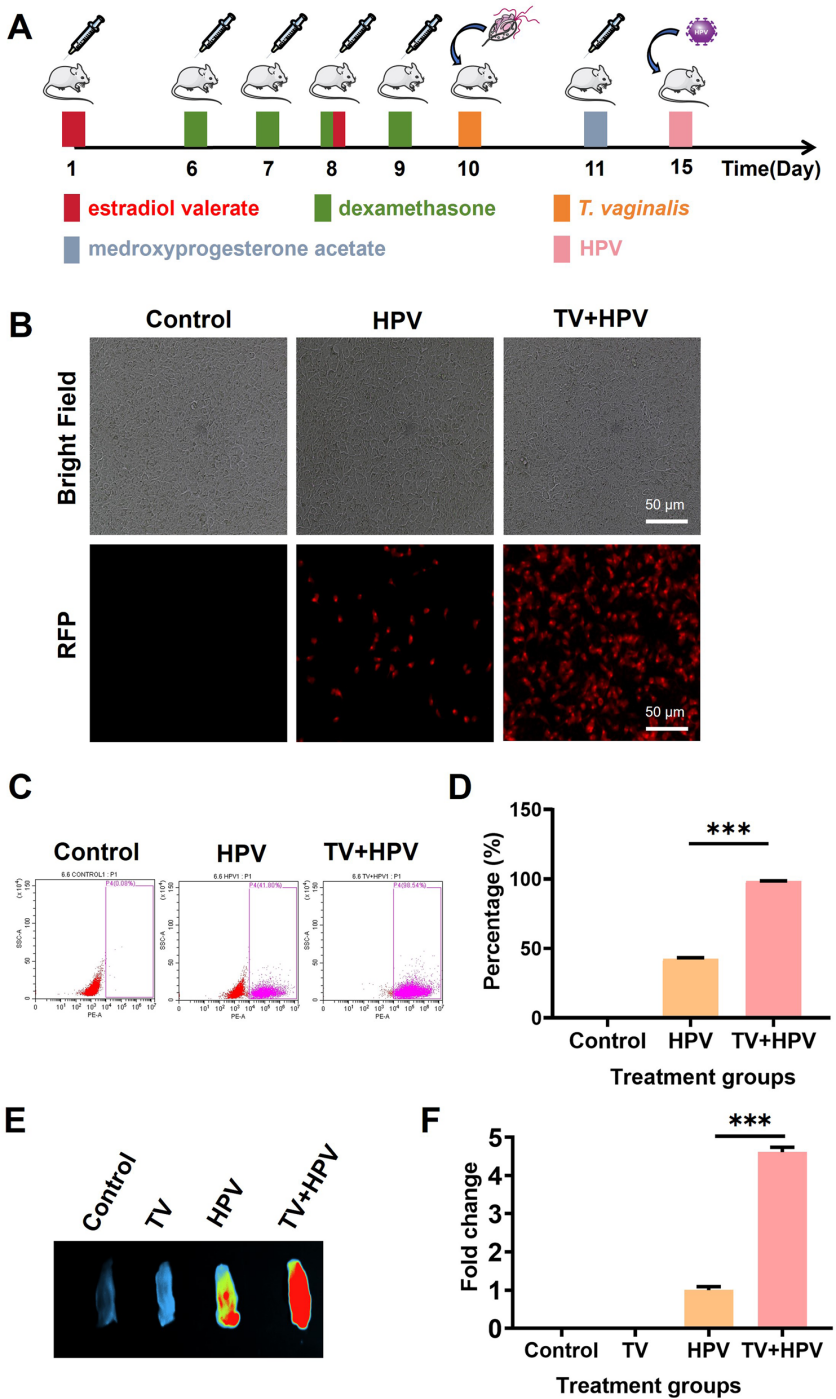
*T. vaginalis* enhanced HPV infection. “Control” represents untreated HaCaT cells or mice, “TV” represents mice infected only with *T. vaginalis*, “HPV” represents HaCaT cells or mice infected only with HPV, and “TV + HPV” represents HaCaT cells or mice infected with both *T. vaginalis* and HPV. The effect of *T. vaginalis* on HPV infection was analyzed by comparing these different treatment groups. **A** Experimental protocol flowchart illustrating the infection timeline in the murine model. **B** Representative fluorescence microscopy images showing HPV infection in HaCaT cells under different treatment conditions. **C** The HPV infection rate in HaCaT cells was detected by flow cytometry. **D** Flow cytometry data was analyzed using GraphPad Prism 9.0 software based on three independent biological replicates. **E** The HPV infection in the vagina of mice was observed by in vivo imaging system. **F** HPV fluorescence intensity in mouse vaginal tissue was quantified using ImageJ, based on three independent biological replicates. HPV: human papillomavirus, TV: *Trichomonas vaginalis*, RFP: red fluorescent protein, and “***”: *P* < 0.001

To investigate the impact of *T. vaginalis* on HPV infection, we employed both in vitro (HaCaT cells) and in vivo (BALB/cA-nu mice) models (Fig. 1). Flow cytometry revealed that co-infection with *T. vaginalis* dramatically increased HPV18 infection rates in HaCaT cells, rising from 42.53 ± 1.10% to 98.39 ± 1.11% (*P* < 0.001; Fig. 1C, D). This enhancement was corroborated in vivo, where vaginal HPV load in mice infected with *T. vaginalis* exhibited a 4.61-fold increase compared to controls (*P* < 0.001; Fig. 1E, F). To ensure that this observed increase was not due to cytotoxic effects or cell death caused by *T. vaginalis*, cell viability was rigorously assessed: Trypan blue staining confirmed that > 93% of HaCaT cells remained viable post-infection (93.06 ± 0.90%, Additional file 6: Fig. S6A), and Calcein AM live-cell staining demonstrated that HPV predominantly co-localized with viable cells (Additional file 6: Fig. S6B).

### *T. vaginalis* upregulates HPV entry receptors CD151 and HSPG2

**Fig. 2:**
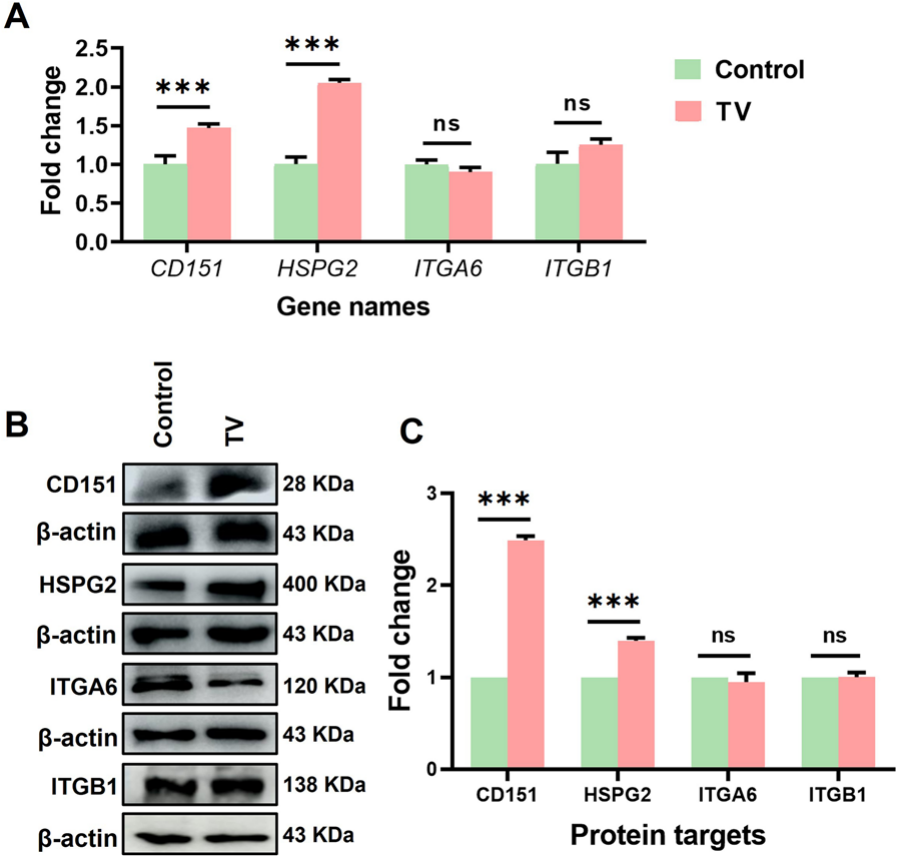
*T. vaginalis* upregulated HPV entry receptors CD151 and HSPG2. “Control” represents untreated HaCaT cells, and “TV” represents HaCaT cells infected with *T. vaginalis*. **A** qPCR analysis of mRNA levels of *HSPG2*, *CD151*, *ITGA6* and *ITGB1* in HaCaT cells, based on three independent biological replicates and three technical replicates per sample; **B** Western blot detection of protein expression levels of HSPG2, CD151, ITGA6 and ITGB1 in HaCaT cells; **C** Quantitative analysis of protein expression levels from panel B using ImageJ software, based on three independent biological replicates. TV: *Trichomonas vaginalis*, CD151: cluster of differentiation 151, HSPG2: heparan sulfate proteoglycan 2, ITGA6: integrin alpha 6, ITGB1: integrin beta 1, ns: no statistical difference, and “***”: *P* < 0.001

To elucidate the mechanistic basis of *T. vaginalis*-mediated facilitation of HPV infection, receptor expression was comprehensively analyzed by qPCR and Western blot. The results showed that *T. vaginalis* infection significantly and selectively upregulated HSPG2 and CD151 at both the mRNA and protein levels, with transcriptional upregulation of 2.05- and 1.47-fold and translational increases of 1.40- and 2.49-fold, respectively (*P* < 0.001; Fig. 2). This selective modulation did not extend to integrins ITGA6 and ITGB1, secondary HPV receptors, whose expression remained unchanged, indicating a targeted effect on the primary viral entry pathway.

### TvAP65 drives HPV entry via receptor activation

**Fig. 3:**
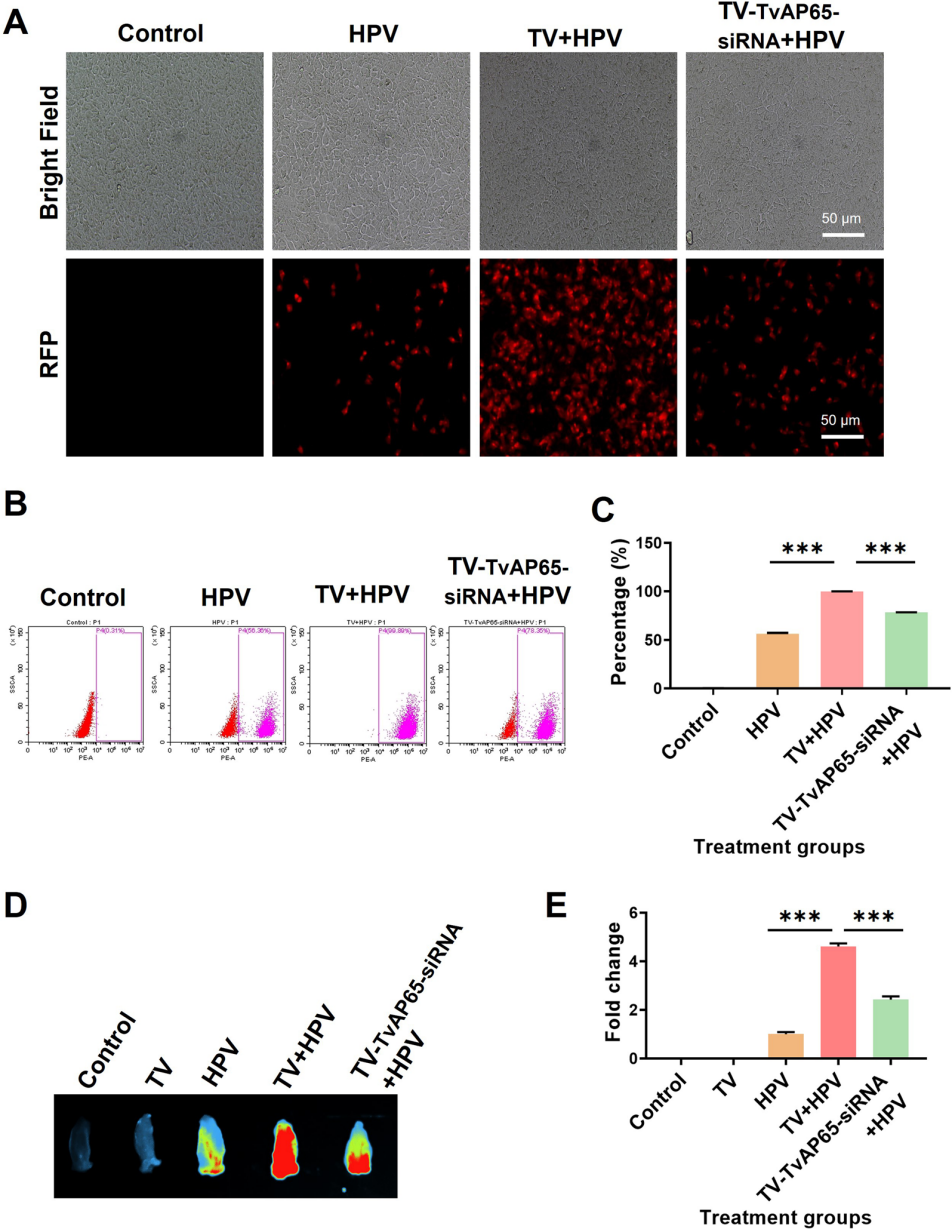
TvAP65 knockdown reversed *T. vaginalis*’s pro-infective effects. “Control” represents untreated HaCaT cells or mice, “TV” represents mice infected only with *T. vaginalis*, “HPV” represents HaCaT cells or mice infected only with HPV, “TV + HPV” represents HaCaT cells or mice infected with both *T. vaginalis* and HPV, “TV-TvAP65-siRNA + HPV” represents HaCaT cells or mice co-infected with *T. vaginalis* in which TvAP65 expression was silenced, together with HPV. Comparison of different treatment groups to assess the role of *T. vaginalis* TvAP65 in modulating HPV infection. **A** Fluorescence microscopy images showing HPV infection in HaCaT cells after TvAP65 knockdown. **B** Flow cytometry analysis of HPV infection rates in HaCaT cells following TvAP65 knockdown. **C** Quantitative analysis of flow cytometry data using GraphPad Prism 9.0, based on three independent biological replicates. **D** In vivo imaging of HPV infection in mice vagina tissue after TvAP65 knockdown. **E** Quantification of HPV fluorescence intensity in mouse vaginas by ImageJ software, based on three independent biological replicates. HPV: human papillomavirus, TV: *Trichomonas vaginalis*, TvAP65: *Trichomonas vaginalis* adhesion protein 65, RFP: red fluorescent protein, and “***”: *P* < 0.001

**Fig. 4:**
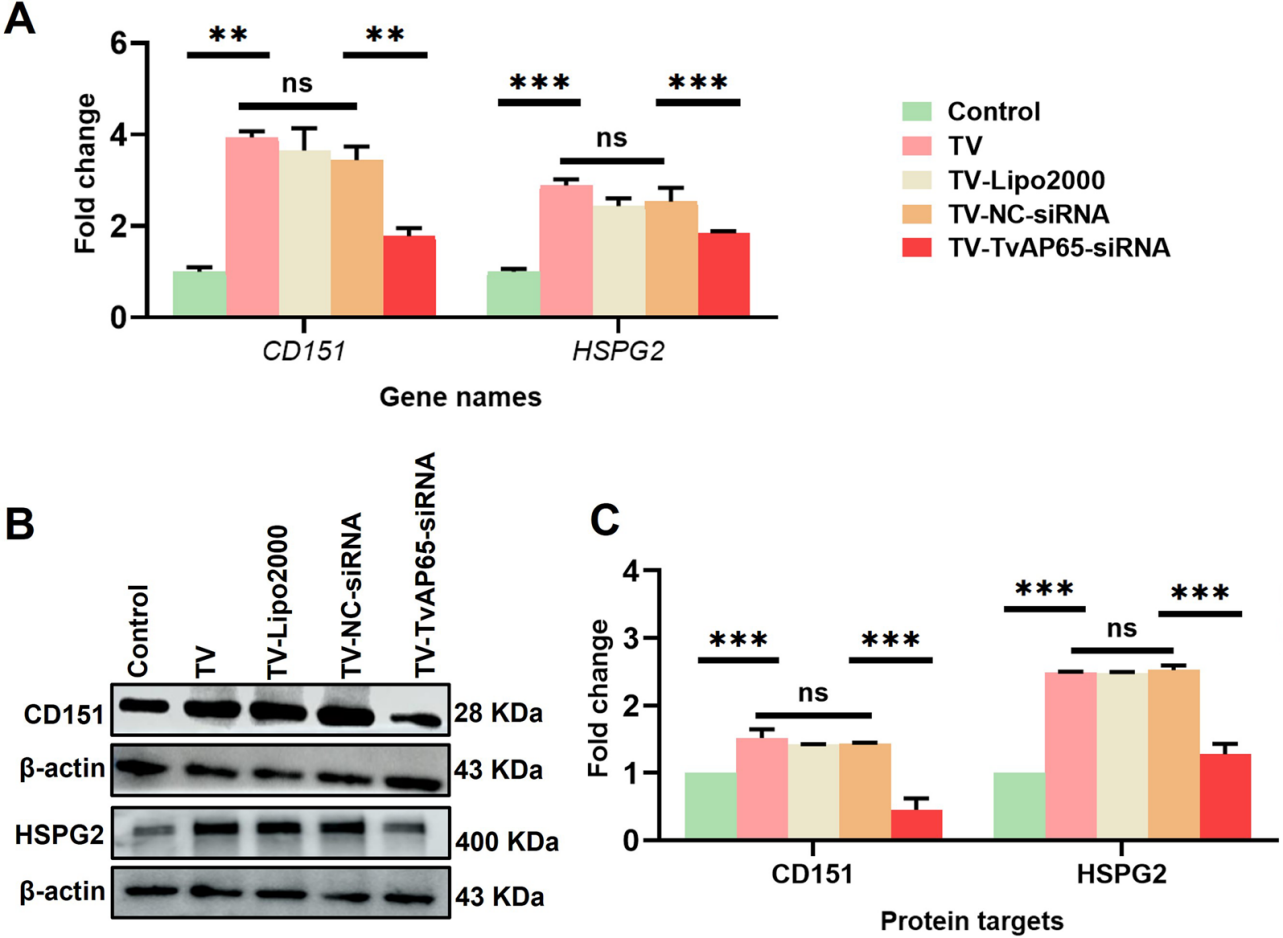
TvAP65 knockdown reduced *T. vaginalis*-induced upregulation of CD151 and HSPG2. “Control” represents untreated HaCaT cells, “TV” represents HaCaT cells infected with *T. vaginalis*, and “TV-TVAP65-siRNA” represents HaCaT cells infected with *T. vaginalis* with downregulated TvAP65 expression, “TV-NC-siRNA” and “TV-Lipo2000” served as siRNA interference control group. **A** qPCR analysis of *HSPG2* and *CD151* mRNA levels in HaCaT cells, based on three independent biological replicates and three technical replicates per sample. **B** Western blot analysis of HSPG2 and CD151 protein expression. **C** Quantification of protein expression levels using ImageJ software, based on three independent biological replicates. TV: *Trichomonas vaginalis*, CD151: cluster of differentiation 151, HSPG2: heparan sulfate proteoglycan 2, TvAP65: *Trichomonas vaginalis* adhesion protein 65, NC: negative control, ns: no statistical difference, “**”: *P* < 0.01, and “***”: *P* < 0.001

**Fig. 5:**
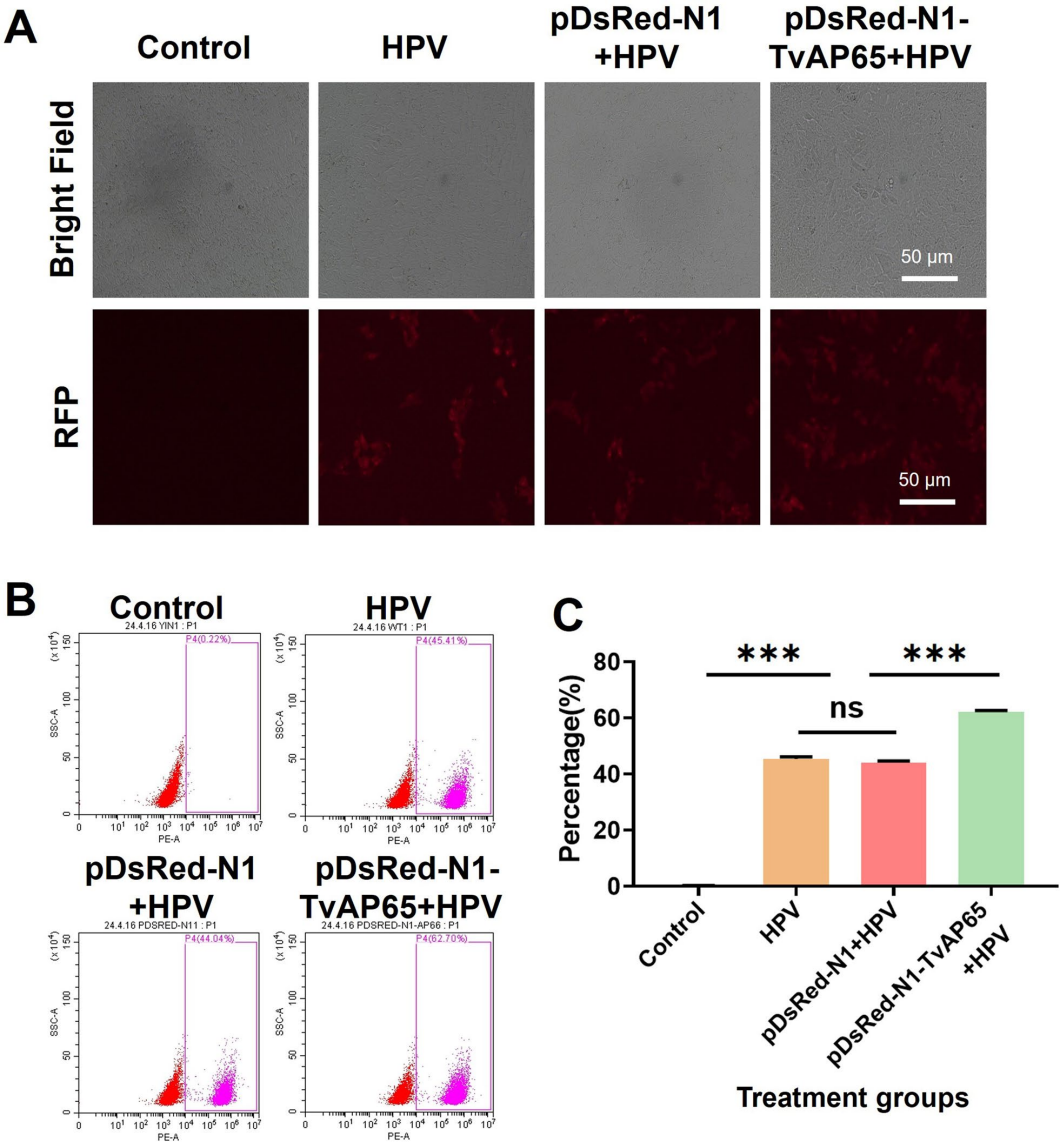
TvAP65 over-expression promoted HPV infection. “Control” represents untreated HaCaT cells, “HPV” represents HaCaT cells infected with HPV, “pDsRed-N1-TvAP65 + HPV” represents HaCaT cells over-expressing TvAP65 and infected with HPV, “pDsRed-N1 + HPV” represents the overexpression vector control group infected with HPV. The comparison among these groups was used to assess the effect of TvAP65 overexpression on HPV infection. **A** Representative fluorescence microscopy images showing HPV infection in HaCaT cells under each treatment condition. **B** Flow cytometry showing HPV infection rate in HaCaT cells over-expressing TvAP65. **C** Statistical analysis of flow cytometry results from three independent biological replicates using GraphPad Prism 9.0. HPV: human papillomavirus, TvAP65: *Trichomonas vaginalis* adhesion protein 65, ns: no statistical difference, RFP: red fluorescent protein, and “***”: *P* < 0.001

**Fig. 6:**
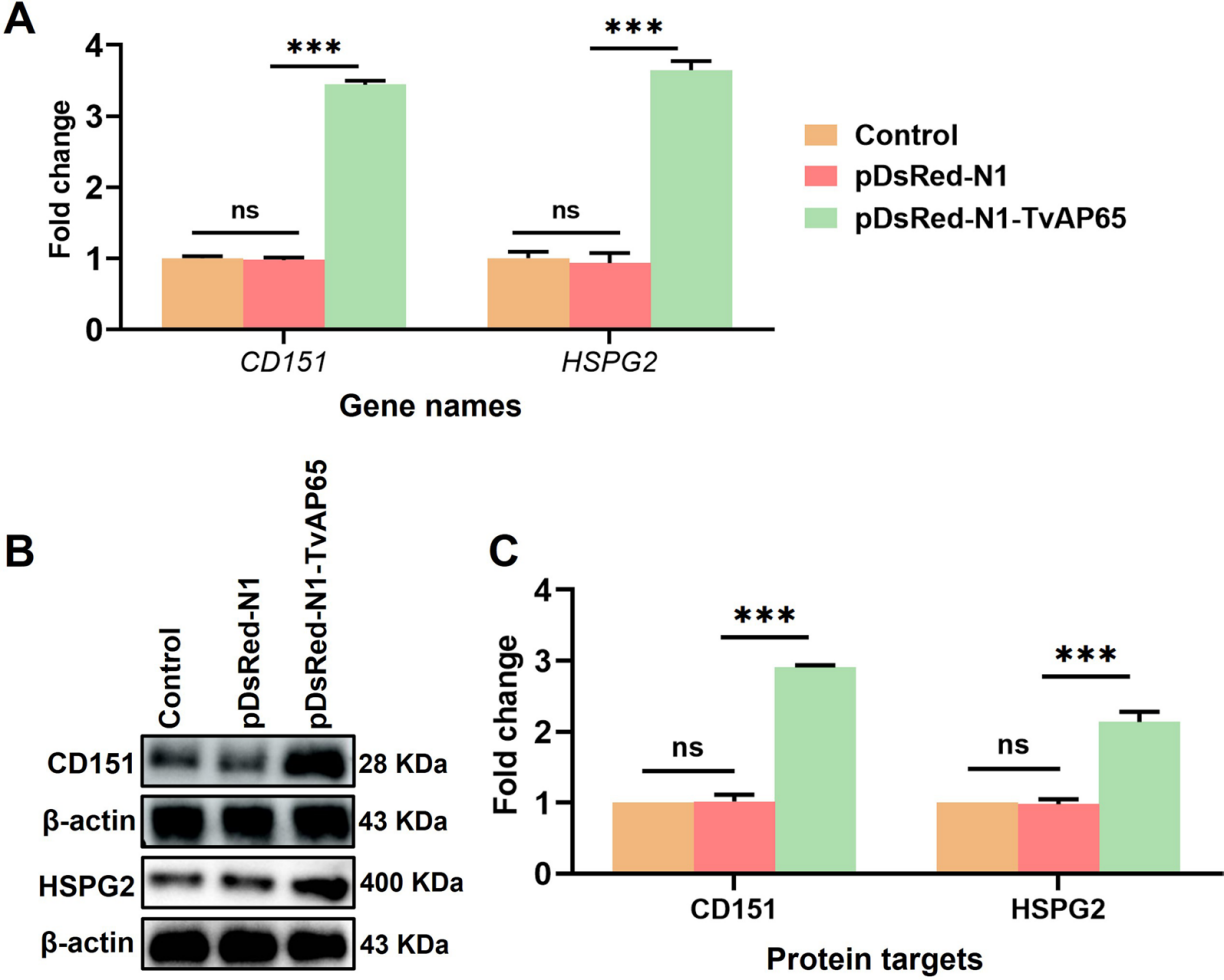
TvAP65 over-expression promoted the expression of CD151 and HSPG2. “Control” represents untreated HaCaT cells, “pDsRed-N1-TvAP65” represents HaCaT cells overexpressing TvAP65, “pDsRed-N1” represents the vector control group. **A** qPCR analysis showing the mRNA levels of *HSPG2* and *CD151* in HaCaT cells, based on three independent biological replicates with three technical replicates per sample. **B** Western blot detection of HSPG2 and CD151 protein expression in HaCaT cells. **C** Quantification of HSPG2 and CD151 protein expression using ImageJ software, based on three independent biological replicates. CD151: cluster of differentiation 151, HSPG2: heparan sulfate proteoglycan 2, TvAP65: *Trichomonas vaginalis* adhesion protein 65, ns: no statistical difference, and “***”:* P* < 0.001

To evaluate the role of TvAP65 in HPV infection, TvAP65 expression was silenced in *T. vaginalis* using siRNA, achieving a 63.48 ± 2.38% reduction in protein level at 72 h (*P* < 0.001; Additional file 7: Fig. S7). TvAP65 knockdown significantly attenuated the pro-infective effect of *T. vaginalis*, reducing HPV infection by 21.76 ± 0.12% in vitro and 1.90-fold in vivo (*P* < 0.001; Fig. 3). This reduction was accompanied by marked downregulation of HSPG2 (3.69-fold) and CD151 (1.95-fold) protein expression (*P* < 0.001; Fig. 4). Conversely, TvAP65 overexpression in HaCaT cells not only enhanced HPV infection by 16.89 ± 0.48% (*P* < 0.001; Fig. 5) but also upregulated HSPG2 and CD151 by 2.14-fold and 2.91-fold, respectively (*P* < 0.001; Fig. 6).

### Synergistic suppression of HPV by dual knockdown

**Fig. 7:**
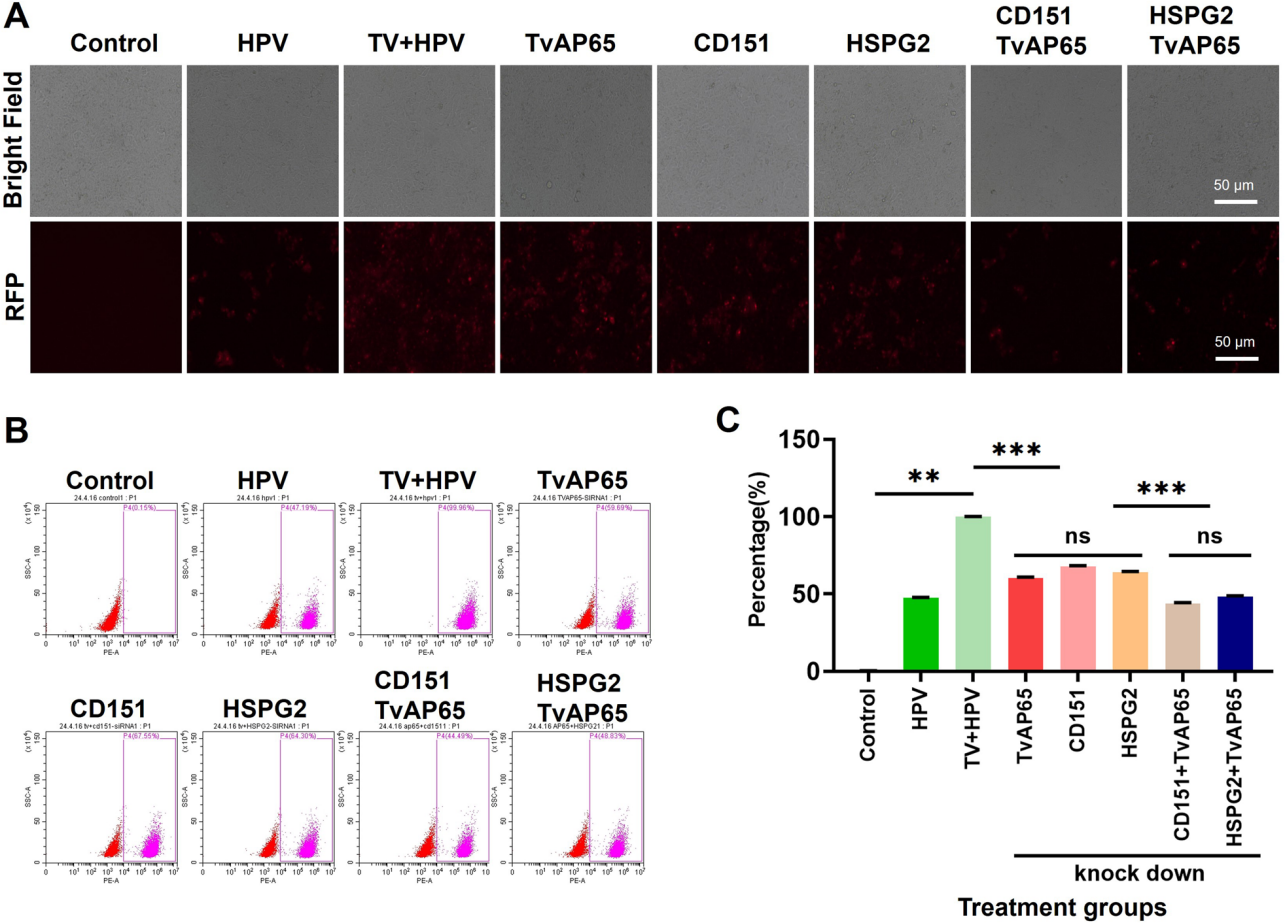
TvAP65 promoted HPV infection by up-regulating CD151/HSPG2 expression. “Control” represents untreated HaCaT cells; “HPV” represents HaCaT cells infected with HPV; “TV + HPV” represents HaCaT cells infected with both *T. vaginalis* and HPV; “TvAP65”, “CD151”, “HSPG2”, “CD151/TvAP65”, and “HSPG2/TvAP65” represent HaCaT cells infected with both *T. vaginalis* and HPV under low-expression of TvAP65, CD151, HSPG2, CD151/TvAP65, or HSPG2/TvAP65, respectively. Comparison of the different treatment groups clarified the mediating role of CD151/HSPG2 in *T. vaginalis* TvAP65-induced enhancement of HPV infection. **A** Representative fluorescence microscopy images showing HPV infection in HaCaT cells following knockdown of the indicated genes. **B** Flow cytometry histograms illustrating HPV infection rates in each knockdown condition. **C** Quantitative analysis of flow cytometry data using GraphPad Prism 9.0, based on three independent biological replicates. HPV: human papillomavirus, TV: *Trichomonas vaginalis*, CD151: cluster of differentiation 151, HSPG2: heparan sulfate proteoglycan 2, TvAP65: *Trichomonas vaginalis* adhesion protein 65, RFP: red fluorescent protein, ns: no statistical difference, “**”: *P* < 0.01, and “***”: *P* < 0.001

To clarify whether the pro-infective effect of TvAP65 is mediated predominantly through CD151 and HSPG2, we performed dual-gene knockdown experiments. Simultaneous silencing of TvAP65 and CD151 resulted in a 56.0 ± 30.27% reduction in HPV infection, which was significantly greater than the effects of silencing TvAP65 alone (39.75 ± 0.42%) or CD151 alone (32.18 ± 0.28%) (*P* < 0.001; Fig. 7). Likewise, co-silencing TvAP65 and HSPG2 led to a 51.70 ± 0.28% decrease in HPV infection, also exceeding the reductions observed with individual knockdowns of TvAP65 (39.75 ± 0.42%) or HSPG2 (36.02 ± 0.34%) (*P* < 0.001; Fig. 7).

### SPCS1 mediates TvAP65-induced HPV receptor upregulation

**Fig. 8:**
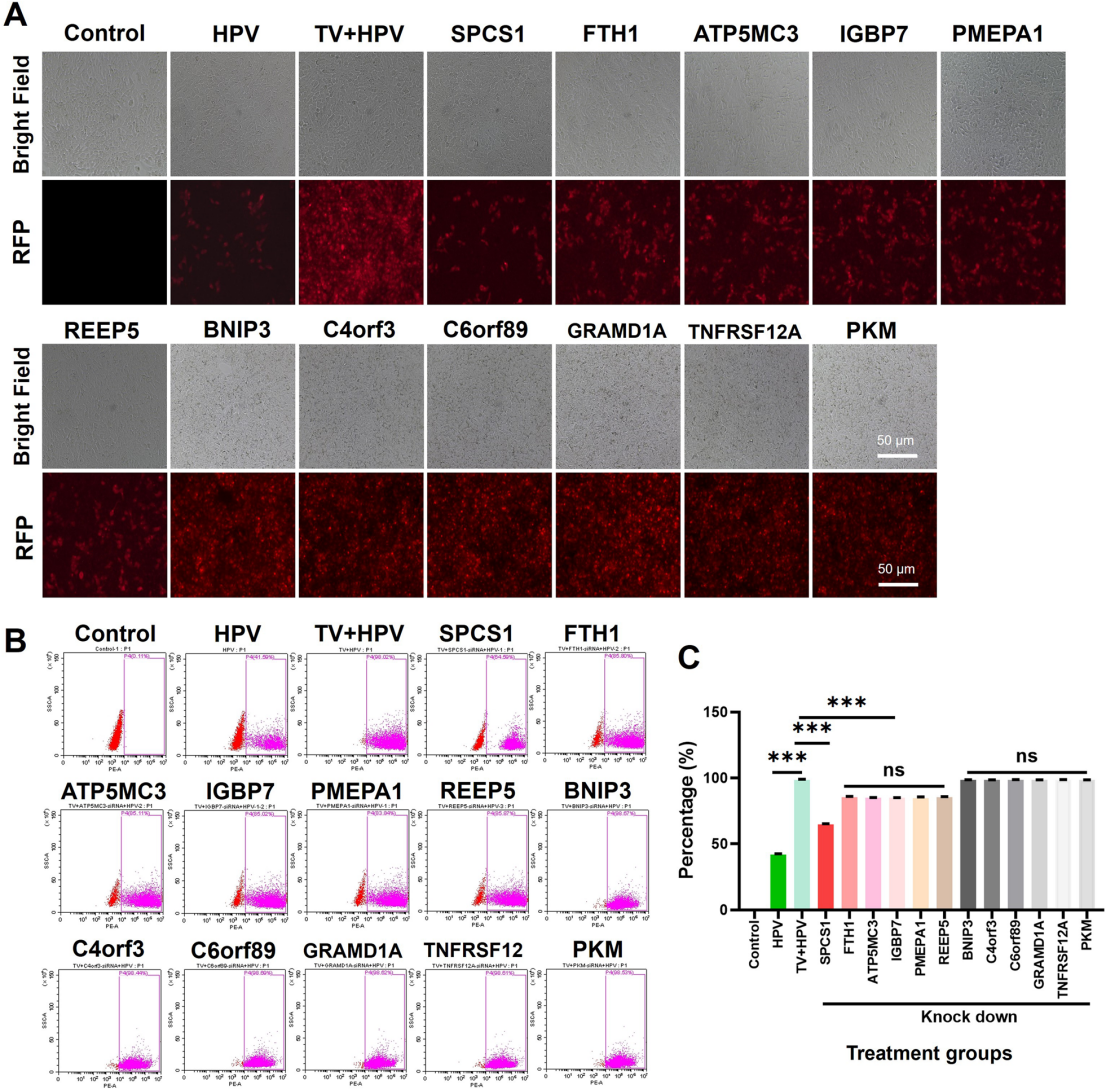
SPCS1 downregulation significantly reduced *T. vaginalis*-induced enhancement of HPV infection. “Control” represents untreated HaCaT cells; “HPV” represents HaCaT cells infected with HPV; “TV + HPV” represents HaCaT cells co-infected with *T. vaginalis* and HPV; “SPCS1”, “FTH1”, “ATP5MC3”, “IGFBP7”, “PMEPA1”, “REEP5”, “BNIP3”, “C4orf3”, “C6orf89”, “GRAMD1A”, “TNFRSF12A”, and “PKM” represent HaCaT cells co-infected with *T. vaginalis* and HPV under knockdown of each respective TvAP65-interacting molecule. Comparisons among different groups were performed to evaluate the impact of TvAP65-interacting molecules on HPV infection. **A** Fluorescence microscopy images showing HPV infection in HaCaT cells after knockdown of the indicated genes. **B** and **C** Flow cytometry quantification of HPV infection rates following gene knockdowns, based on three independent biological replicates. HPV: human papillomavirus, TV: *Trichomonas vaginalis*, SPCS1: signal peptidase complex subunit 1, RFP: red fluorescent protein, ns: no statistical difference, and “***”: *P* < 0.001. All other gene names (FTH1, ATP5MC3, IGBP7, PMEPA1, REEP5, BNIP3, C4orf3, C6orf89, GRAMD1A, TNFRSF12A, PKM) are official gene symbols according to the National Center for Biotechnology Information Gene database and are presented in their standard abbreviated form

**Fig. 9:**
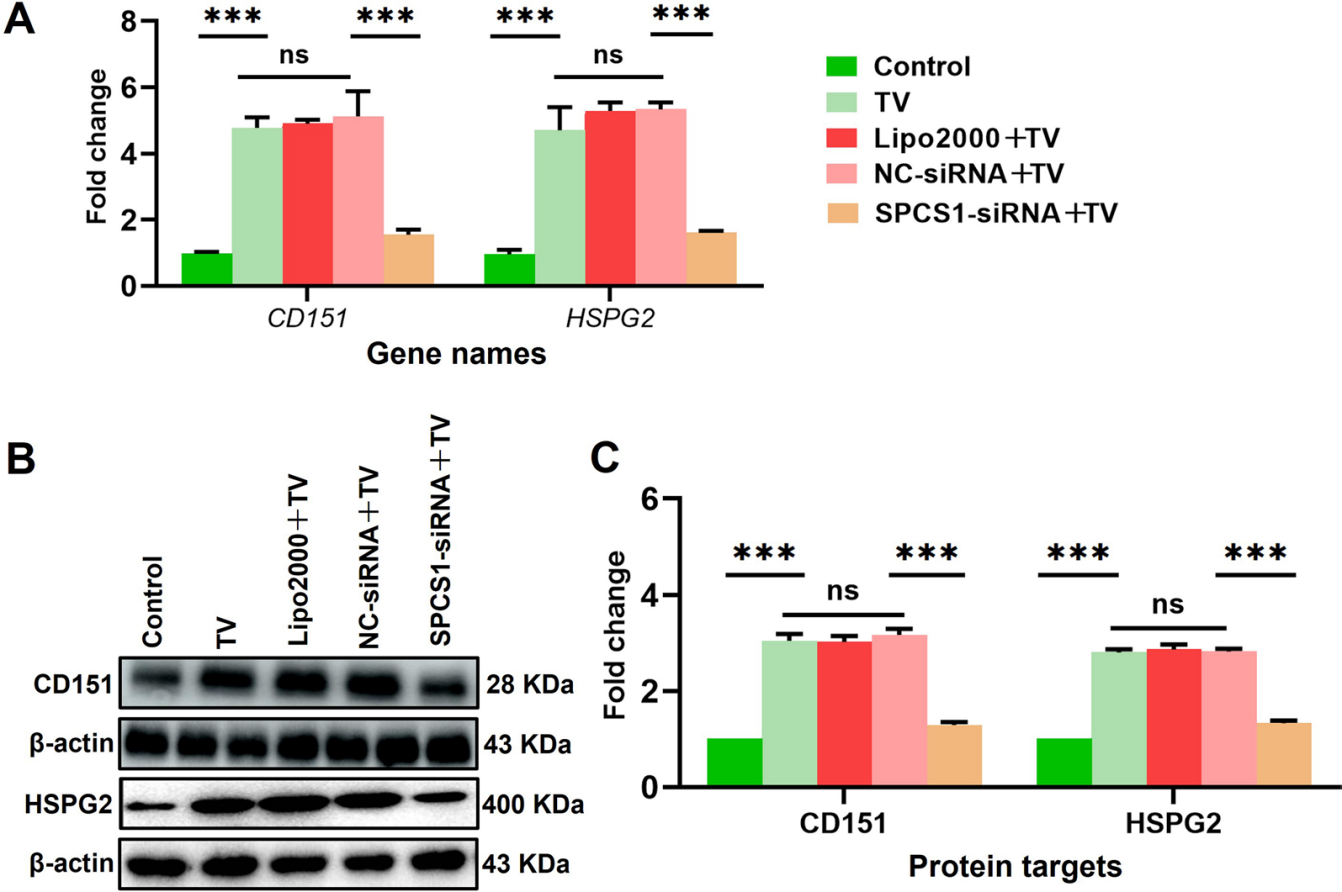
SPCS1 downregulation significantly reduced *T. vaginalis*-induced upregulation of CD151 and HSPG2. “Control” represents untreated HaCaT cells, “TV” represents HaCaT cells infected with *T. vaginalis*, and “SPCS1-siRNA + TV” represents that HaCaT cells infected with *T. vaginalis* underunder SPCS1 knockdown. “NC-siRNA + TV” and “Lipo2000 + TV” served as controls for negative control siRNA and Lipofectamine 2000 treatments, respectively. **A** qPCR was used to quantify *HSPG2* and *CD151* mRNA levels in HaCaT cells, with three biological replicates and three technical replicates per sample. **B** Western blotting was performed to detect protein expression levels of HSPG2 and CD151 in HaCaT cells. **C** Protein expression levels of HSPG2 and CD151 were quantified using Image J, based on three independent biological replicates. TV: *Trichomonas vaginalis*, CD151: cluster of differentiation 151, HSPG2: heparan sulfate proteoglycan 2, SPCS1: signal peptidase complex subunit 1, NC: negative control, ns: no statistical difference, and “***”: *P* < 0.001

**Fig. 10:**
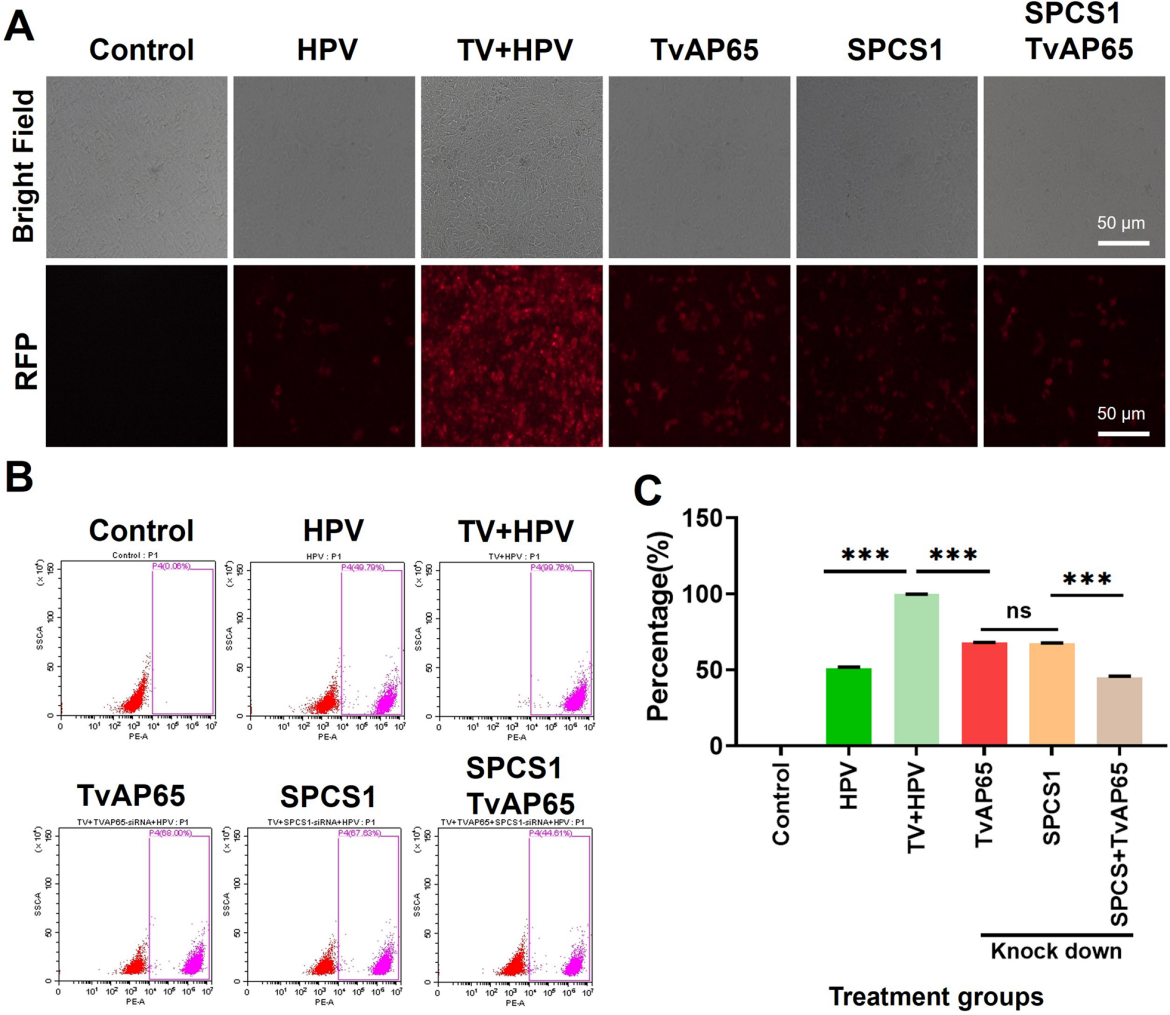
TvAP65 promoted HPV infection through interaction interacting with SPCS1. “Control” represents untreated HaCaT cells; “HPV” represents HaCaT cells infected with HPV; “TV + HPV” represents HaCaT cells co-infected with *T. vaginalis* and HPV; “TvAP65”, “SPCS1”, and “SPCS1/TvAP65” represent HaCaT cells co-infected with *T. vaginalis* and HPV under knockdown of TvAP65, SPCS1, or both genes, respectively. **A** HPV infection in HaCaT cells was visualized by fluorescence microscopy following gene knockdown. **B** and **C** HPV infection in HaCaT cells was evaluated by flow cytometry after gene downregulation, using three independent biological replicates. HPV: human papillomavirus, TV: *Trichomonas vaginalis*, TvAP65: *Trichomonas vaginalis* adhesion protein 65, SPCS1: signal peptidase complex subunit 1, ns: no statistical difference, and “***”: *P* < 0.001

To pinpoint host factors responsible for mediating the effects of TvAP65, we performed an siRNA screen targeting 12 candidate proteins known to interact with TvAP65. Of these, SPCS1 knockdown most potently attenuated HPV infection (33.61 ± 0.40% reduction, *P* < 0.001; Fig. 8) and significantly suppressed *T. vaginalis*-driven CD151/HSPG2 upregulation (Fig. 9). Strikingly, dual knockdown of TvAP65 and SPCS1 further suppressed HPV infection by 54.64 ± 0.39% (*P* < 0.001, Fig. 10), a reduction significantly greater than that achieved by either knockdown alone (Fig. 10), indicating a functional synergy between TvAP65 and SPCS1. Furthermore, molecular docking predicted a direct binding interface between TvAP65 and SPCS1, and this physical interaction was experimentally validated by Co-IP in 293 T cells (Additional file 8: Fig. S8).

## Discussion

The global burden of sexually transmitted infections (STIs) is staggering, with *T. vaginalis* and HPV representing two of the most prevalent pathogens [29]. Over 376 million new STI cases are reported annually among individuals aged 15–49, with co-infections significantly increasing morbidity and cancer risk [2]. Epidemiological studies consistently link *T. vaginalis* infection to heightened HPV susceptibility and cervical dysplasia [30], yet the molecular basis of this synergy remain poorly understood. Our study bridges this gap by uncovering a novel mechanism: *T. vaginalis* hijacks its adhesion protein TvAP65 to upregulate the HPV entry receptors CD151 and HSPG2 via the host factor SPCS1, thereby creating a permissive niche for viral invasion.

Pathogen co-infections often exploit overlapping niches, as seen with *Gardnerella vaginalis*, which enhances HPV infection by degrading protective mucins [31, 32], and *Lactobacillus* spp., which promote viral clearance via IFN-γ induction [33]. Parasites like *T. vaginalis* may manipulate host microenvironments through cysteine proteases or substrate remodeling to facilitate viral persistence [34–36]. However, prior studies have largely focused on inflammation or immune suppression as drivers of HPV-*T. vaginalis* synergy [19–21]. Our findings challenge this conventional view by demonstrating that *T. vaginalis* directly enhances HPV tropism through receptor upregulation, independent of its cytotoxic effects—a mechanism distinct from those of other STI-associated pathogens.

To initiate infection, HPV engages specific membrane receptors. We found that *T. vaginalis* infection selectively upregulates HSPG2 and CD151 (mRNA: 2.05-/1.47-fold; protein: 1.40-/2.49-fold), while leaving ITGA6 and ITGB1 expression unchanged (Fig. 2). This specificity suggests *T. vaginalis* targets early viral attachment steps rather than later endocytic events, actively remodeling the host cell surface to elevate HSPG2 and CD151, thereby creating a more permissive environment for HPV attachment and entry and enhancing viral tropism. Central to this process is TvAP65, a malic enzyme homolog critical for parasite adhesion [23, 24, 37]. Knockdown of TvAP65 led to a 21.76 ± 0.12% reduction in HPV infection (*P* < 0.001), accompanied by a 3.69-fold and 1.95-fold decrease in HSPG2 and CD151 protein expression, respectively (Figs. 3–4), while overexpression enhanced both receptor levels (Figs. 5–6). These bidirectional functional experiments confirm that TvAP65 is both necessary and sufficient for *T. vaginalis*-mediated HPV receptor upregulation and infection. Further dual-gene knockdown experiments targeting TvAP65 together with either CD151 or HSPG2 revealed that co-silencing TvAP65 and HSPG2 or CD151, led to a more pronounced reduction in HPV infection compared with knocking down each gene individually (Figs. 7). These results suggest that TvAP65 predominantly enhances HPV entry through upregulation of CD151 and HSPG2, while alternative or compensatory pathways play only a minor role in this process.

To elucidate the underlying host pathways, we screened 12 TvAP65 interactors. SPCS1 emerged as the key mediator. Knockdown of SPCS1 alone reduced HPV infection by 33.61 ± 0.40% (*P* < 0.001) and significantly suppressed *T. vaginalis*-driven CD151/HSPG2 upregulation (Figs. 8–9). Strikingly, dual knockdown of TvAP65 and SPCS1 synergistically suppressed infection (54.64 ± 0.39% reduction, *P* < 0.001; Fig. 10), surpassing the effects of individual knockdowns. These results indicate that TvAP65 hijacks SPCS1 to enhance the surface expression of CD151 and HSPG2, thereby facilitating HPV entry. We propose a model whereby the TvAP65-SPCS1 interaction facilitates trafficking of HSPG2 and CD151 to the cell surface, enhancing HPV binding (Graphical Abstract image). This mechanism diverges from previously characterized TvAP65 interactions (e.g., BNIP3-mediated adhesion [38–41]), highlighting functional versatility of *T. vaginalis* in subverting host pathways and establishing SPCS1 as a potential therapeutic target to disrupt HPV-*T. vaginalis* synergy.

Our findings redefine the *T. vaginalis*-HPV synergy beyond inflammation-driven damage. By upregulating HSPG2/CD151, *T. vaginalis* establishes a feedforward loop that amplifies HPV entry, particularly in persistent infections. This mechanism may account for the reported 6.5-fold increase in HPV16 risk among *T. vaginalis*-positive women [13] and the limited efficacy of HPV vaccines in co-infected populations [42].

However, several limitations should be noted. First, our in vivo model utilized immunocompromised BALB/cA-nu mice, which may underestimate the contribution of adaptive immunity in shaping *T. vaginalis*-HPV co-infection dynamics. Second, although HaCaT cells are widely used in HPV research for their stable growth and susceptibility, they lack some innate immune components and epithelial differentiation seen in primary tissues. Future studies employing immunocompetent animal models, primary human cervical epithelial cells, or organotypic raft cultures are essential to validate these findings in more physiologically relevant contexts.

Additionally, clinical cohort studies are needed to correlate TvAP65/SPCS1 expression levels with HPV persistence and cervical dysplasia in *T. vaginalis*-positive patients. Exploring SPCS1’s broader role in other *T. vaginalis*-associated pathologies, such as preterm birth or HIV co-infection, could uncover shared mechanisms of host subversion. Addressing these gaps will deepen our understanding of the TvAP65-SPCS1 axis and its therapeutic implications.

Our discovery of the TvAP65-SPCS1-CD151/HSPG2 axis offers new avenues for therapeutic intervention. Targeting this pathway—such as with inhibitors disrupting TvAP65-SPCS1 interaction or neutralizing TvAP65 antibodies—may prevent or reduce co-infection by *T. vaginalis* and HPV, thereby lowering cervical cancer risk. This mechanistic insight could guide development of dual-action therapeutics targeting both parasitic and viral infections in the female reproductive tract.

## Conclusions

We unveil a tripartite axis—*TvAP65-SPCS1-CD151/HSPG2*—through which *T. vaginalis* exacerbates HPV infection. This mechanism transcends traditional paradigms of inflammation or immune suppression, offering a framework for mechanistically targeted interventions. As HPV vaccines coverage gaps persist and *T. vaginalis* resistance escalates, targeting this axis may help reduce cervical cancer risk in high-burden populations.

## Supplementary Information

Additional file 1.

Additional file 2.

Additional file 3.

Additional file 4.

Additional file 5.

Additional file 6.

Additional file 7.

Additional file 8.

## Data Availability

The original contributions presented in the study are included in the article. Further inquiries can be directed to the corresponding author.

## Abbreviations

ANOVA: Analysis of variance
CD151: Cluster of differentiation 151
CMC: Carboxymethylcellulose
Co-IP: Co-immunoprecipitation
DMEM: Dulbecco's modified eagle medium
FBS: Fetal bovine serum
GAPDH: Glyceraldehyde 3-phosphate dehydrogenase
HIV: Human immunodeficiency virus
HPV: Human papillomavirus
HSPG2: Heparan sulfate proteoglycan 2
ITGA6: Integrin alpha 6
ITGB1: Integrin beta 1
NC: Negative control
OD: Optical density
PBS: Phosphate-buffered saline
RFP: Red fluorescent protein
RNAi: RNA interference
SPCS1: Signal peptidase complex subunit 1
SPF: Specific pathogen-free
TV: *Trichomonas vaginalis*
TvAP65: *Trichomonas vaginalis* Adhesion protein 65
TYM: Trypticase-yeast extract-maltose
