# Supplementary figures and images for: Trichomonas vaginalis adhesion protein 65 facilitates human papillomavirus entry via SPCS1-mediated upregulation of CD151 and HSPG2 in keratinocyte lineage
Source: Infect Dis Poverty. 2025 Nov 6;14:114. doi: 10.1186/s40249-025-01381-x (PMC12590808; doi:10.1186/s40249-025-01381-x)

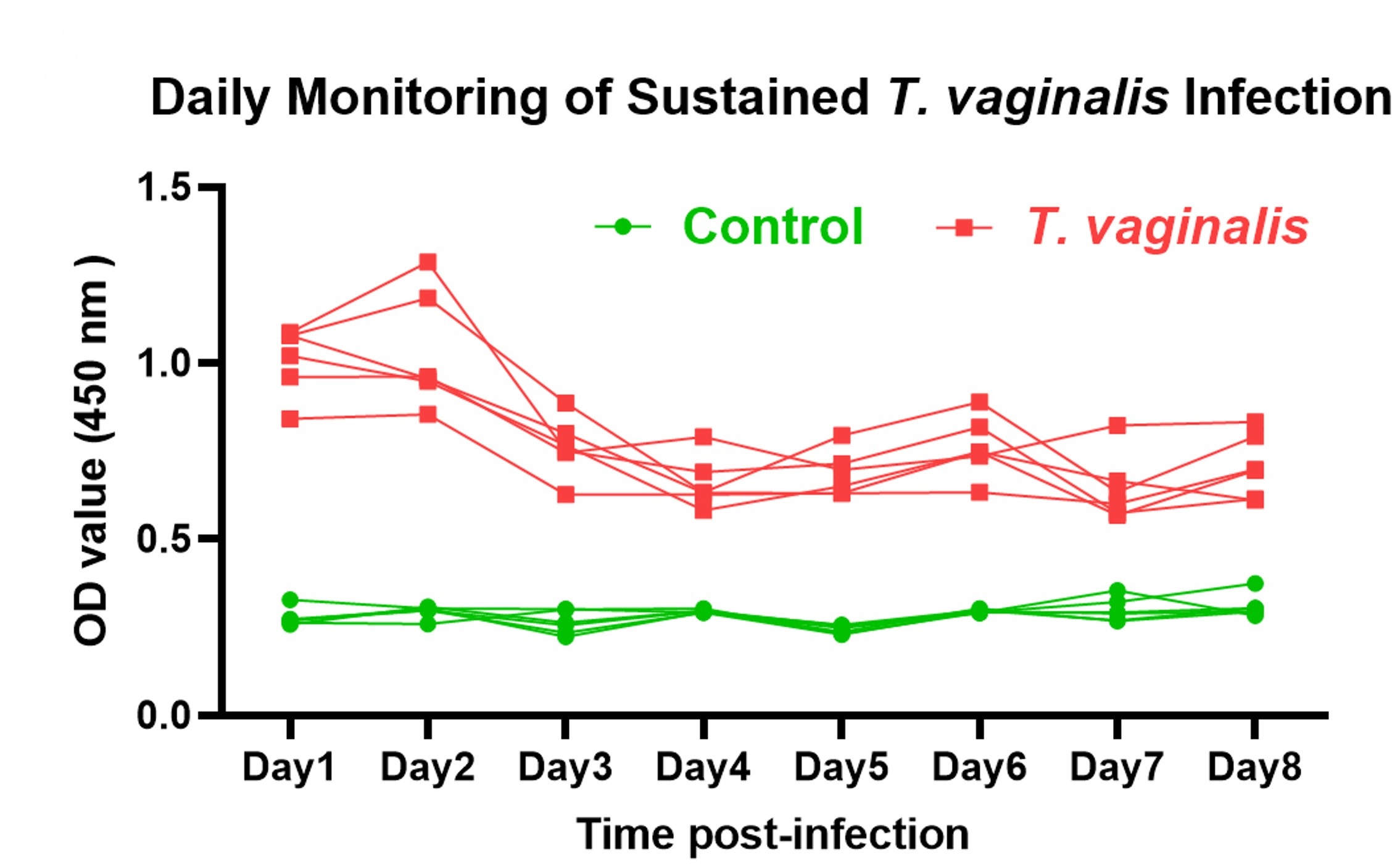

Supplement: Supplementary file 1 — Additional file 1. [file 40249_2025_1381_MOESM1_ESM.jpg]

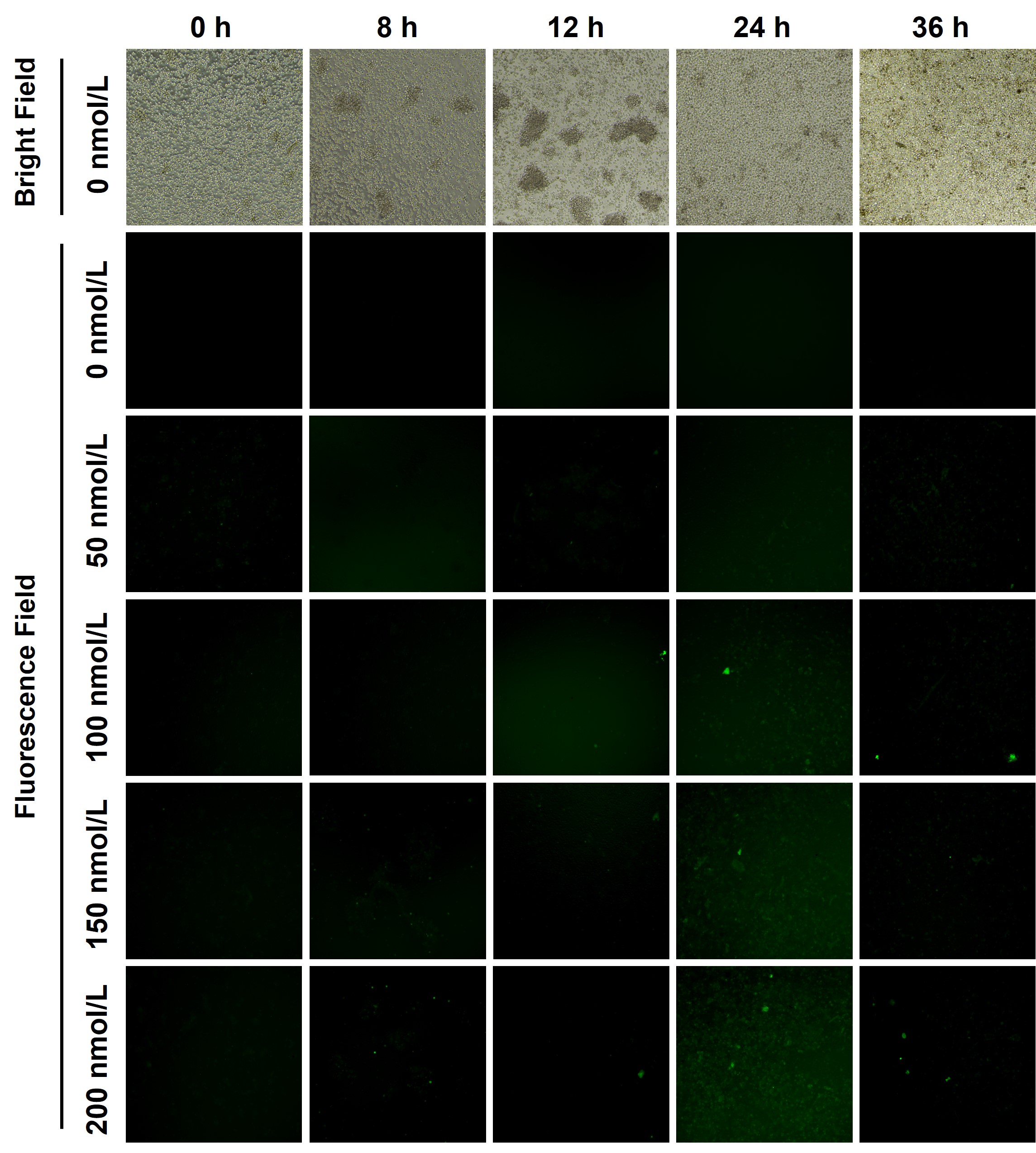

Supplement: Supplementary file 2 — Additional file 2. [file 40249_2025_1381_MOESM2_ESM.jpg]

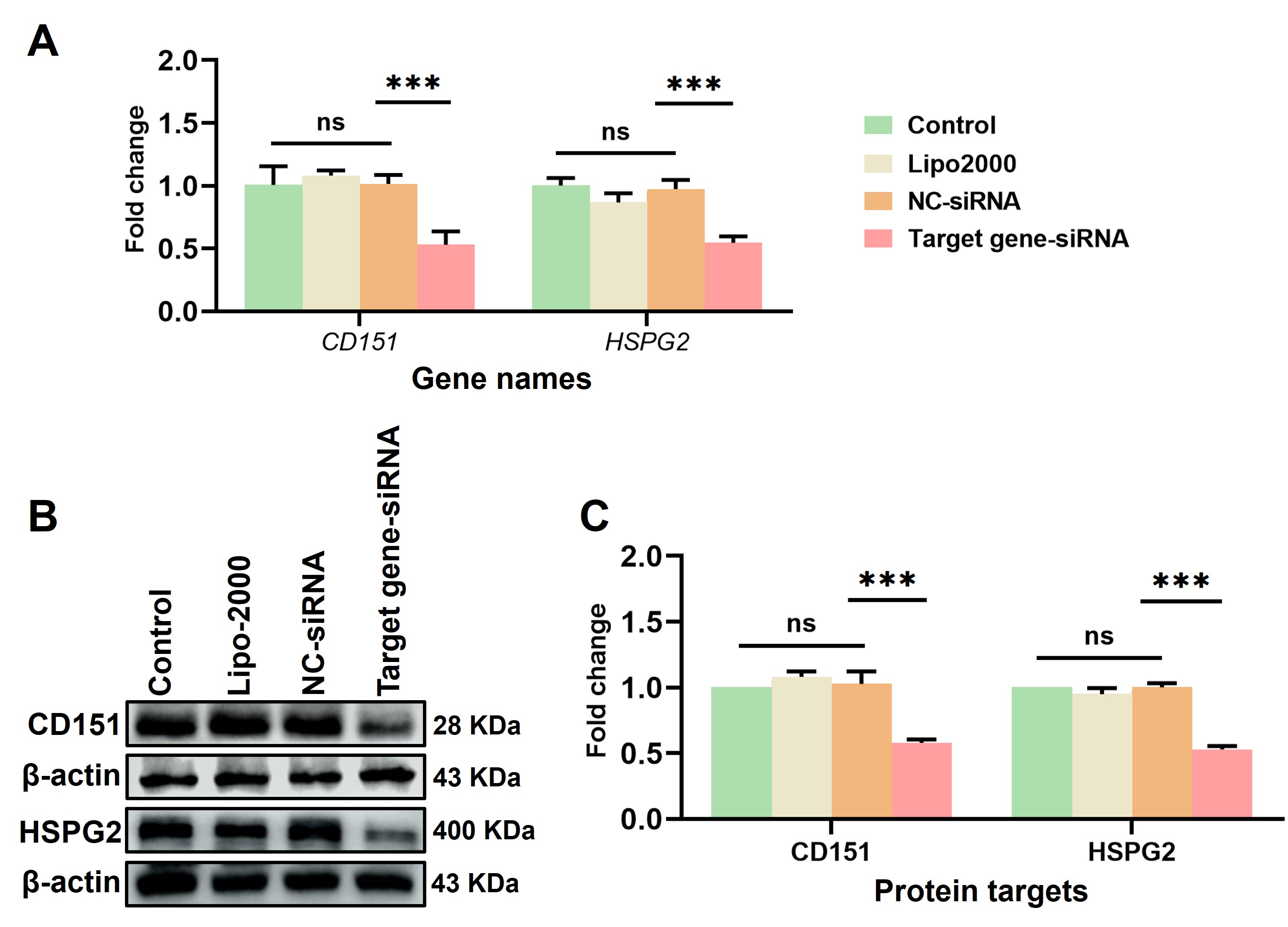

Supplement: Supplementary file 3 — Additional file 3. [file 40249_2025_1381_MOESM3_ESM.jpg]

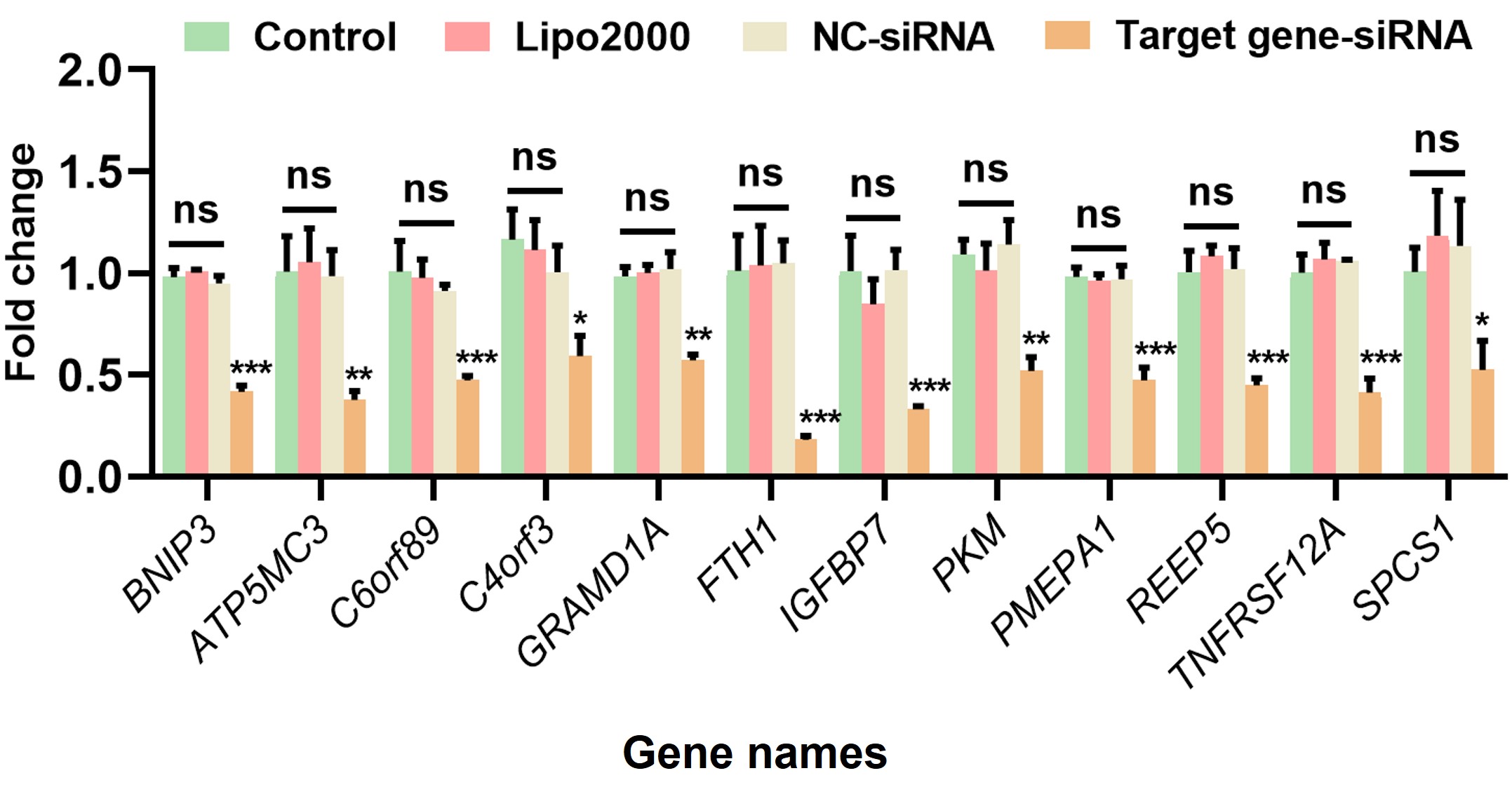

Supplement: Supplementary file 4 — Additional file 4. [file 40249_2025_1381_MOESM4_ESM.jpg]

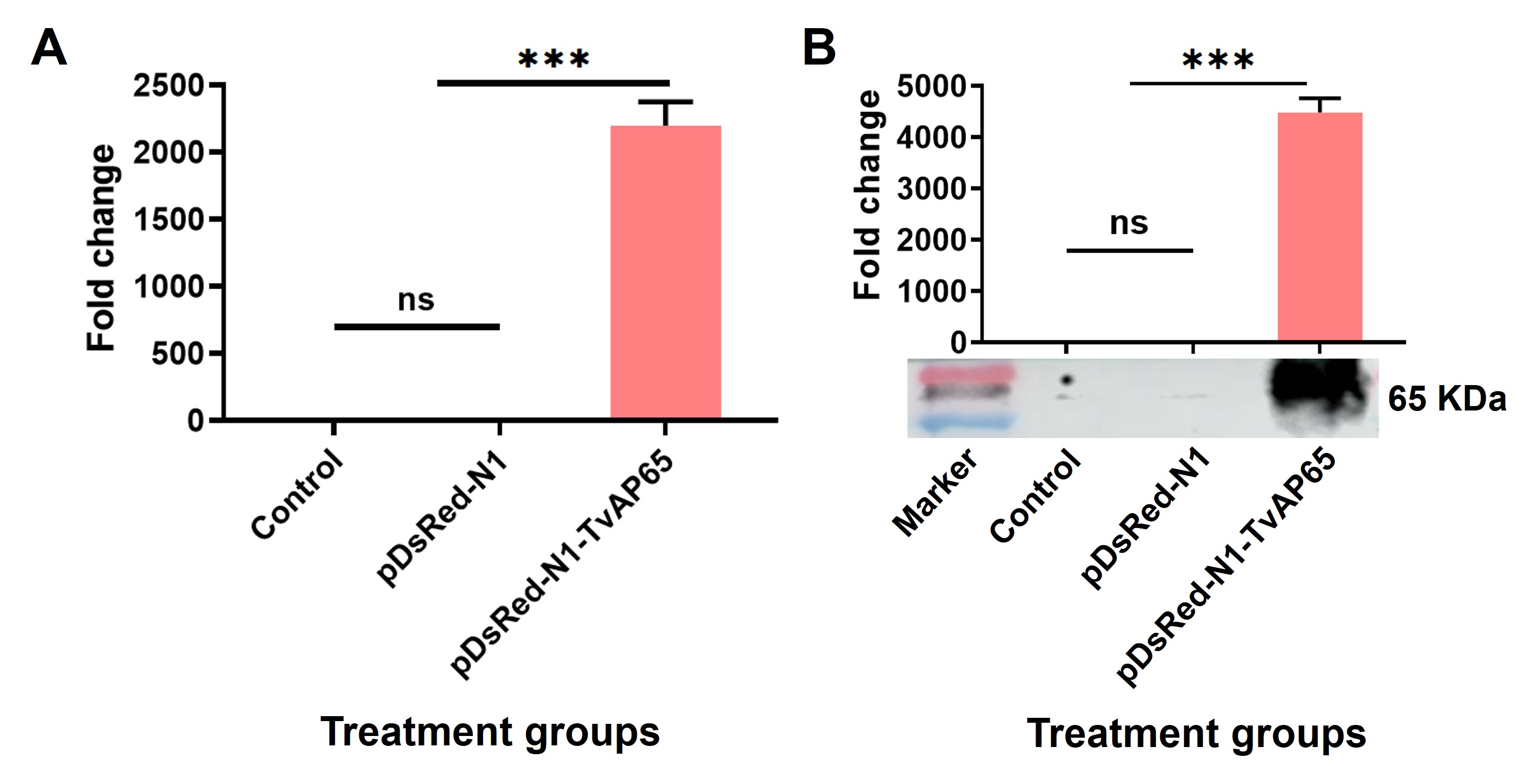

Supplement: Supplementary file 5 — Additional file 5. [file 40249_2025_1381_MOESM5_ESM.jpg]

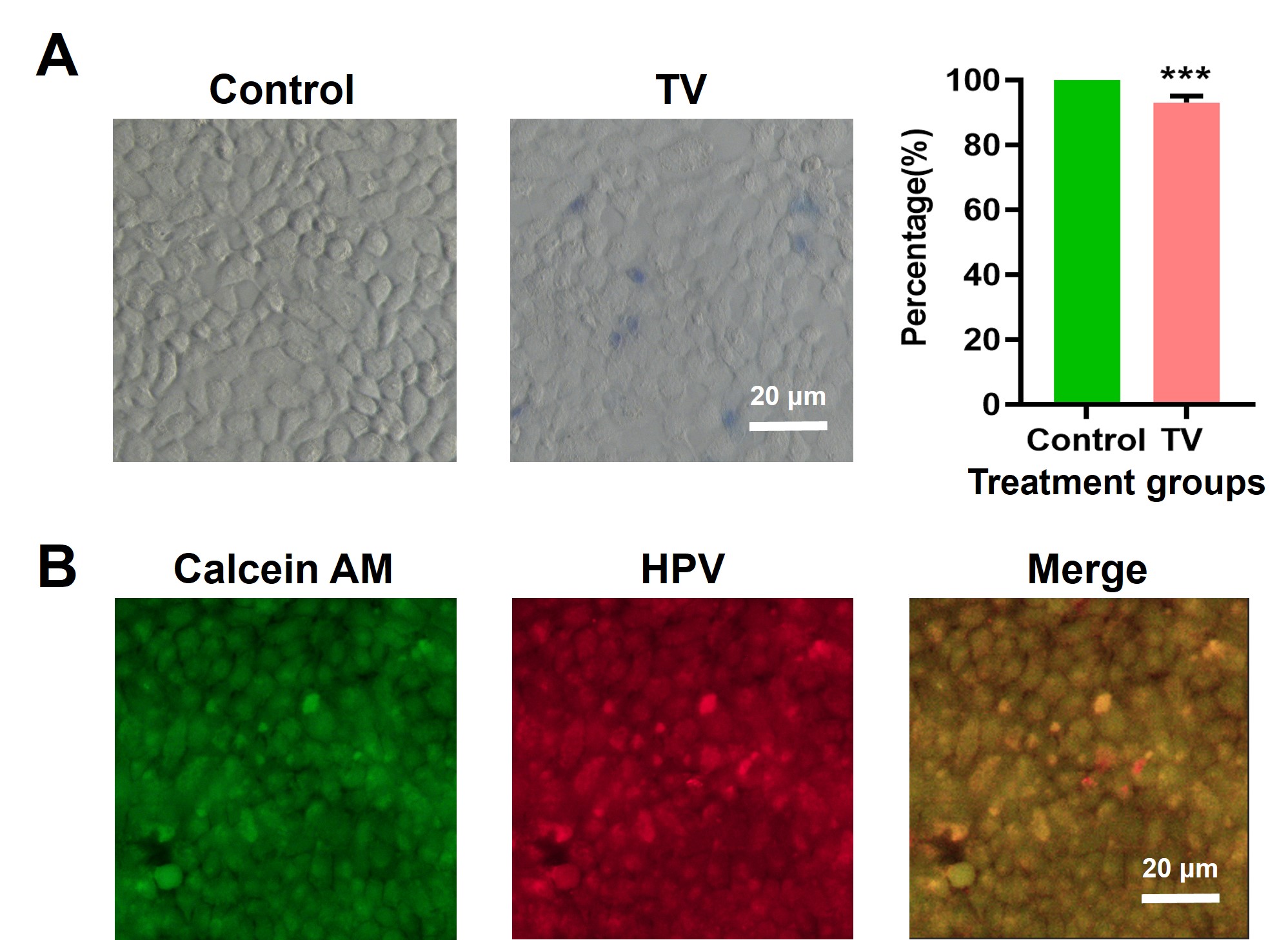

Supplement: Supplementary file 6 — Additional file 6. [file 40249_2025_1381_MOESM6_ESM.jpg]

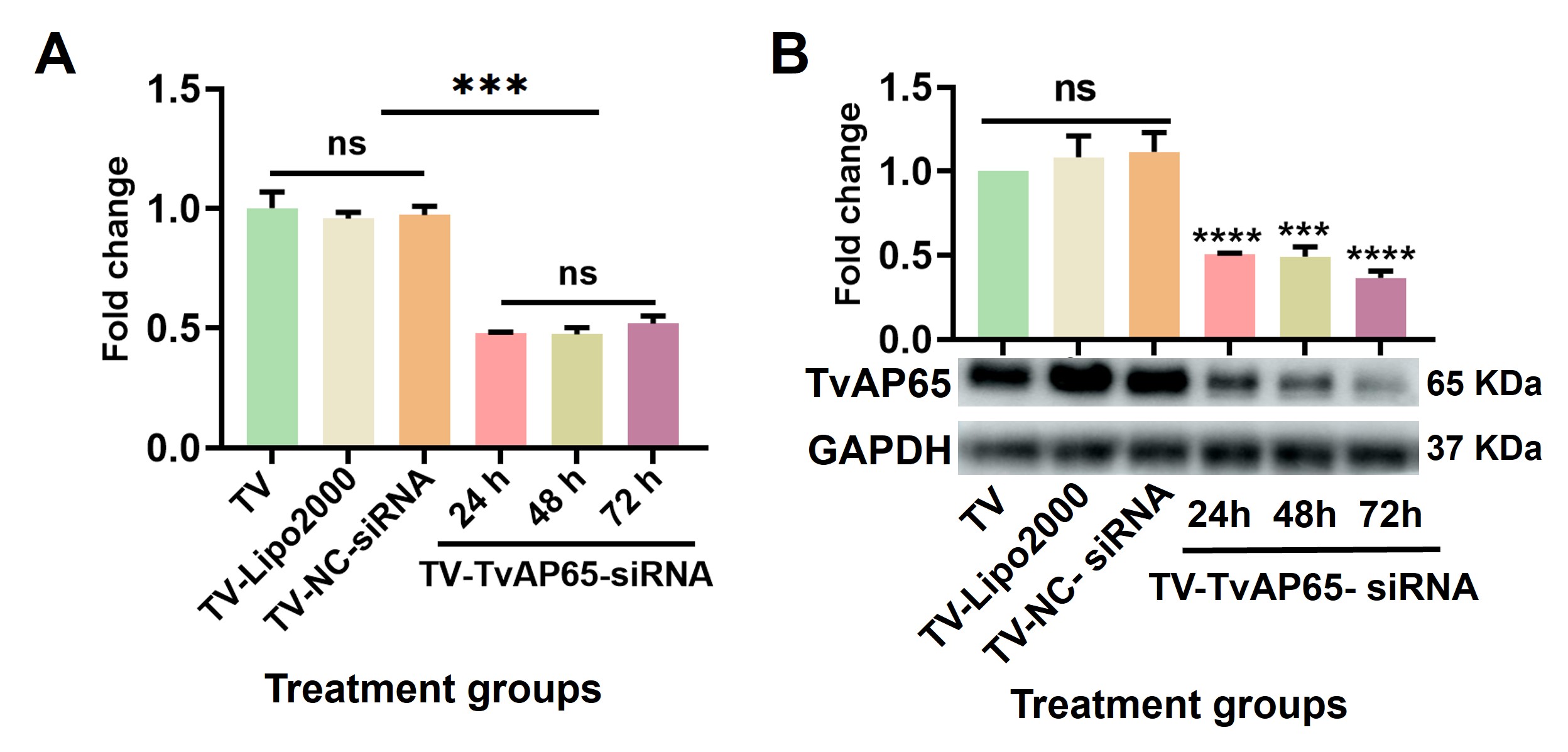

Supplement: Supplementary file 7 — Additional file 7. [file 40249_2025_1381_MOESM7_ESM.jpg]

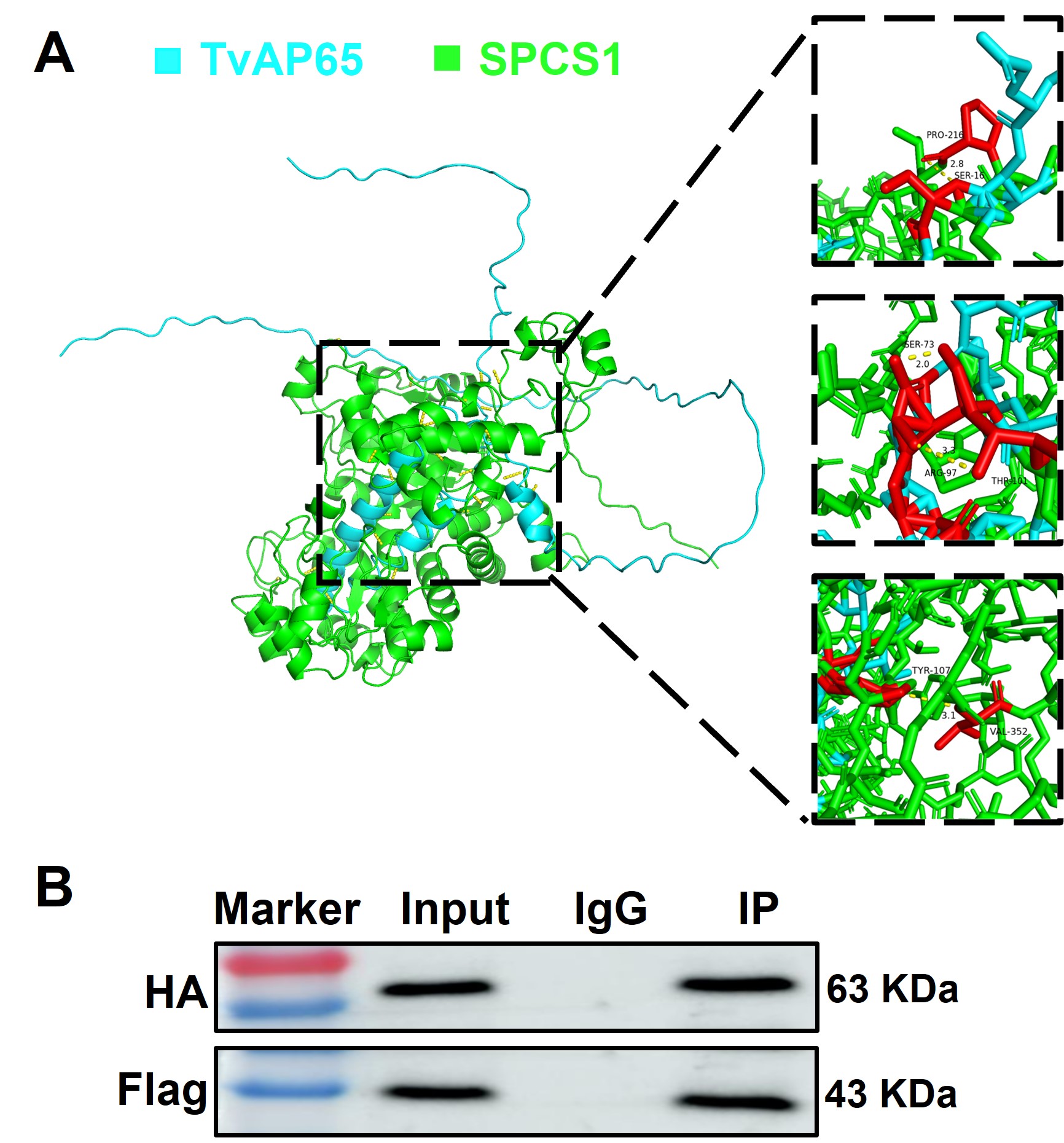

Supplement: Supplementary file 8 — Additional file 8. [file 40249_2025_1381_MOESM8_ESM.jpg]
